# Environmental fluctuations and their effects on microbial communities, populations and individuals

**DOI:** 10.1093/femsre/fuaa068

**Published:** 2020-12-18

**Authors:** Jen Nguyen, Juanita Lara-Gutiérrez, Roman Stocker

**Affiliations:** Institute for Environmental Engineering, Department of Civil, Environmental and Geomatic Engineering, ETH Zürich, 8093 Zürich, Switzerland; Microbiology Graduate Program, Massachusetts Institute of Technology, Cambridge, MA 02139, USA; Institute for Environmental Engineering, Department of Civil, Environmental and Geomatic Engineering, ETH Zürich, 8093 Zürich, Switzerland; Institute for Environmental Engineering, Department of Civil, Environmental and Geomatic Engineering, ETH Zürich, 8093 Zürich, Switzerland

**Keywords:** population dynamics, single cell, microbial responses, changing environments, microbial evolution, microbial physiology

## Abstract

From the homeostasis of human health to the cycling of Earth's elements, microbial activities underlie environmental, medical and industrial processes. These activities occur in chemical and physical landscapes that are highly dynamic and experienced by bacteria as fluctuations. In this review, we first discuss how bacteria can experience both spatial and temporal heterogeneity in their environments as temporal fluctuations of various timescales (seconds to seasons) and types (nutrient, sunlight, fluid flow, etc.). We then focus primarily on nutrient fluctuations to discuss how bacterial communities, populations and single cells respond to environmental fluctuations. Overall, we find that environmental fluctuations are ubiquitous and diverse, and strongly shape microbial behavior, ecology and evolution when compared with environments in which conditions remain constant over time. We hope this review may serve as a guide toward understanding the significance of environmental fluctuations in microbial life, such that their contributions and implications can be better assessed and exploited.

## Teaser

The spatial and temporal heterogeneity of microbial habitats exposes microbes to a broad range of environmental fluctuations, which drive a broad range of microbial responses that vary with fluctuation timescale and level of microbial organization (communities, populations and individuals).

## INTRODUCTION

This review will demonstrate that environmental fluctuations are ubiquitous, diverse and strongly shape microbial behavior, ecology and evolution. We first elaborate several examples of fluctuations in different environmental systems, in effort to illustrate the sources of environmental fluctuations ubiquitous across all microbial habitats. We then summarize a diversity of observed microbial responses to fluctuations (in nutrient, light, water availability, etc.) that vary across scales of organization (communities, populations, individuals) and highlight important directions in which current research is lacking.

We specifically discuss direct responses to environmental fluctuations. The physiological responses of microbes to single shifts in environmental conditions are extensively reviewed elsewhere (Csonka 1989; Potts 1994; Paul *et al*. 2004). Here, we focus directly on fluctuations (i.e. series of environmental shifts) and their effects on microbial activity. The studies reviewed highlight fluctuations as an important characteristic to our understanding of microbial life, because microbes in fluctuations can display strikingly different behaviors than those responding to single shifts. We illustrate these differences by examining microbial responses to environmental fluctuations at different scales of biological organization. At the community level, fluctuations have been theorized to influence species diversity by fluctuating the availability of different niches faster than species can go extinct. In practice, some microbial communities indeed fluctuate with the environment in composition (Desai *et al*. 2016) and activity (Marchant *et al*. 2017), yet some are surprisingly robust. At the population level, microbes may evolve faster in fluctuating environments than steady ones and employ bet-hedging strategies to endure fluctuations (Cooper and Lenski 2000). At the single-cell level, growing microbes are classically understood to alter their physiology toward the physiological steady state associated with their immediate environmental conditions, yet new work demonstrates that cells instead have fluctuation-adapted physiologies distinct from those characterized in steady environments (Nguyen *et al*. 2020).

Overall, we aim to provide an overview of some of the environmental fluctuations that occur in microbial habitats and describe whether and how these fluctuations affect microbes depends on the relative timescale of fluctuations and the timescale of microbial responses (Table S1, Supporting Information). We draw upon examples from diverse environmental and biological systems to emphasize how the relationship between environmental and biological timescales can be used to understand and study microbial systems in complex and dynamic environments.

## ENVIRONMENTAL FLUCTUATIONS ARE DIVERSE AND PERVASIVE ACROSS MICROBIAL HABITATS

**Figure 1.**
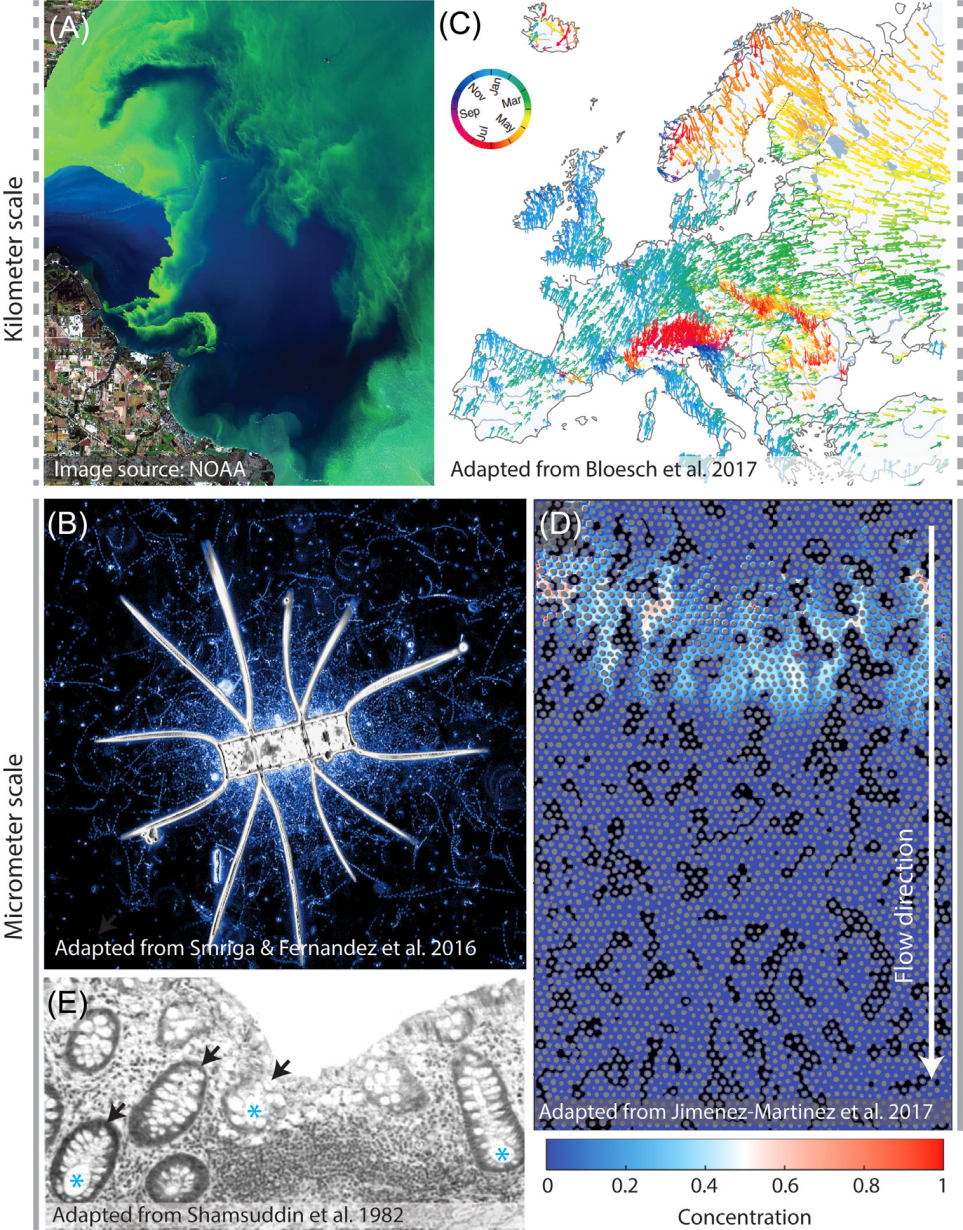
Environmental fluctuations occur across length scales and timescales in diverse habitats. **(A)** Kilometer-scale algal bloom on Western Lake Erie. Courtesy of NOAA (https://oceanservice.noaa.gov/facts/hab-solutions.html). **(B)** A microscale nutrient hotspot is created as a marine diatom secretes metabolites into the local environment. Chemotactic bacteria accumulate around the hotspot, as shown by the time projection (Smriga et al. 2016). **(C)** A temporal map of European river flooding events, which bring water on kilometer scales to local soils (Blöschl *et al*. 2017). Colors indicate time of year and arrows indicate how yearly events change over time as the climate changes. **(D)** Microscale image of chemicals seeping into a soil-like porous medium. The void spaces between soil grains (gray) are filled with either air (black) or water (blue): the water saturation and pore size affect how fluid and chemicals are transported through the medium. Chemical heterogeneities (colorbar) arise due to the formation of preferential paths (Jiménez‐Martínez et al. 2017). White arrow indicates flow direction, as chemical seeps into soil. **(E)** Microscale image of the gut epithelium and its 100–500-µm-sized crypts (black arrows) (Shamsuddin, Phelps and Trump 1982). Each crypt is lined by mucus-secreting cells and this mucus contributes to maintain strong gradients in oxygen, antimicrobials and flow, accessible to resident bacteria (blue asterisks).

No natural microbial habitats are truly steady; rather, environmental fluctuations are pervasive characteristics of microbial life. Even conditions with controlled temperature, light and fluid mixing will change as microbes consume and secrete compounds into their surroundings (Parulekar *et al*. 1986; Sezonov, Joseleau-Petit and d'Ari 2007). In sparse environments with low cell density (e.g. the oligotrophic ocean), the secretions of single cells create micron-sized nutrient hotspots of higher than background metabolite concentrations (Taylor and Stocker 2012; Stocker 2015). Conversely, large-scale inputs (e.g. kilometer-scale nutrient runoffs or intermittent upwellings) can induce episodic blooms of algal communities or populations (Heisler *et al*. 2008; Guseva and Feudel 2020) (Fig. 1).

A prime example of an environment characterized by pervasive fluctuations is the ocean. Some fluctuations are daily. For example, in shallow water sediments, the oxic zone expands during sunlight hours as a result of the activity of photosynthetic microorganisms and shrinks during the night (Fenchel 2002). Some other fluctuations occur at faster timescales, often through the heterogeneous spatial structure of microscale parcels of water. Nutrients in the ocean, for example, are not uniformly distributed but rather arise as localized hotspots (Azam and Malfatti 2007; Stocker 2012), often on length scales on the order of 100 µm (Stocker 2015). Metabolites exuded around live phytoplankton (the phycosphere; Seymour *et al*. 2017) and released by lysing cells (Smriga *et al*. 2016), sinking particles of organic matter (Fenchel 2002) and excretions from nearby protozoa (Blackburn, Fenchel and Mitchell 1998) all form local hotspots of nutrient that are orders of magnitude richer than background concentrations. Chemotactic bacteria can traverse the nutrient gradients sourced from these hotspots within timescales of seconds to minutes (Smriga *et al*. 2016) (Fig. 1). Hence, bacterial motility or other forms of relative motion (e.g. settling due to gravity) convert spatial heterogeneity into temporally dynamic nutrient signals. In other words, a single motile bacterium experiences the microscale spatial heterogeneity of nutrient as temporal fluctuations in nutrient concentration. Diffusion can dissipate 100 µm hotspots within 10–20 min (Blackburn, Fenchel and Mitchell 1998; Smriga *et al*. 2016) and turbulence can reshape hotspots into thinner filaments that dissipate even faster (Taylor and Stocker 2012). The ephemeral nature of microscale hotspots and their patchy distribution together contribute to a highly dynamic environment that microorganisms experience as fluctuations.

The fractured spatial structure of soils is a major source of environmental fluctuations for subsurface microorganisms. Variations in soil grain size (<0.25–2 mm) produce variations in pore size (i.e. the void space between grains), which in turn produce variations in pore accessibility to fluid flow (Jiménez-Martínez *et al*. 2017). Soil pores are generally filled with varied proportions of water and air, which further accentuate spatial variability in fluid transport (Fig. 1). Because water facilitates chemical diffusion and transport, heterogeneities in fluid flow give rise to chemical heterogeneities, such as gradients in nutrients and oxygen (Or *et al*. 2007). In sandy sediments, oxygen concentrations can fluctuate on minute timescales as the flow of water fluctuates with changing currents and sediment movements (Billerbeck 2006; Ahmerkamp *et al*. 2015). Fluctuations can also occur due to the sudden release of chemicals into the soil (Vorholt 2012). In the rhizosphere, the area immediately surrounding plant roots, the sudden input of labile exudates from roots can trigger a ‘hot moment’, a short pulse of microbial activity (i.e. increased rate of nutrient decomposition) lasting from minutes to hours—evidence of a microscale nutrient hotspot (Kuzyakov and Blagodatskaya 2015). Fluctuations in soil environments also occur at large spatial scales and long timescales. In humid tropical forests, the combination of high net primary productivity, warm temperatures and abundant rainfall produces fluctuations in redox potential on timescales of hours to days (Pett-Ridge and Firestone 2005; DeAngelis *et al*. 2010); and in arid regions plagued by summer droughts, the water availability in soils, or water saturation, fluctuates across months (Placella, Brodie and Firestone 2012) (Fig. 1). Overall, as in the ocean, the heterogeneous distribution of physical, chemical and biological factors in space and time exposes microbes to a vast diversity of fluctuations in soils.

The mammalian gut is yet another microbial habitat in constant change. Bacteria inhabiting the gut experience changes in nutrient source and concentration as a function of the composition and frequency of meals taken by the host (Carmody *et al*. 2015). Changes in host feeding times (e.g. such as those associated with night shifts or jet lag) alter the relative abundances of different microbial members in the host microbiome (Thaiss *et al*. 2014; Zarrinpar *et al*. 2014). Individual microbes likely experience their host's meals as fluctuations at smaller spatial scales, as the gut is a mechanically active environment. Contractions in the gut lining occur at frequencies of 7–20 min^−1^ in the small intestine and 2–13 min^−1^ in the colon (Gayer and Basson 2009), producing peristaltic waves that can travel at several millimeters per second (Hardcastle and Mann 1968). These contractions contribute to a mean flow velocity of ∼20 µm s^−1^ in the gut as well as fluid mixing, which even creates some backward flow (Cremer *et al*. 2016). Intestinal mixing and flow create a fluctuating environment for bacteria associated with the gut mucosa. Bacterial movement, either through bacterial motility or transport by the fluid flow within and between gut compartments, gives rise to temporal fluctuations as cells traverse through the various scales of heterogeneities in the gut. For example, the fine spatial structure in the colon epithelium (i.e. microscopic intestinal folds) (Fig. 1) and mucus contributes to radial gradients in nutrients, oxygen, fluid flow, immune system factors and bacterial diversity (Donaldson, Lee and Mazmanian 2016; Tropini *et al*. 2017). Longitudinal gradients await bacteria that traverse the different compartments of the gut and their different nutrient and physicochemical conditions. The small intestine has higher levels of oxygen and labile nutrients, whereas the colon is less acidic and predominantly contains nutrients in the form of dietary fibers (Pereira and Berry 2017).

Across diverse habitats, temporal and spatial heterogeneities in the environment translate into environmental fluctuations for bacteria. These fluctuations are diverse in type (i.e. nutrient, sunlight, fluid flow) and timescale (i.e. seconds, hours, seasons). Some fluctuations occur at a generally predictable frequency, as is the case with daily changes in light (Cohen and Golden 2015) and seasonal changes in temperature (Lodge, McDowell and McSwiney 1994; Kuzyakov and Blagodatskaya 2015). Others occur sporadically, as is the case for nutrient patches in the ocean (Blackburn, Fenchel and Mitchell 1998) and soil (Kuzyakov and Blagodatskaya 2015). Environmental fluctuations can be gradual, like the changes in the amount of daily sunlight across the year at higher latitudes, or sharp and drastic, like the first rainfall after a dry summer in arid ecosystems (Placella, Brodie and Firestone 2012).

**Figure 2.**
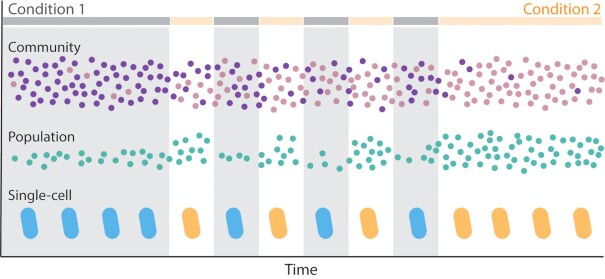
Community, population and single-cell scales of microbial response to fluctuations. Microbial communities, populations and single cells respond to environmental fluctuations through a variety of processes. A community experiencing fluctuations between Condition 1 and Condition 2 (represented by the broken horizontal gray and orange lines) can shift in community composition between the steady states associated with either condition. The purple species dominates the community when the environment is steadily Condition 1, whereas the pink species dominates under steady Condition 2. The relative abundance of these species fluctuates under fluctuating environmental conditions, as the interactions between species change with the environment. A population can change in size with the environment, as illustrated by the population of green cells, which grows faster in Condition 2 than in Condition 1. Finally, single cells can regulate their physiology in response to the immediate environment. Upon sensing Condition 1, a cell may induce gene expression to produce proteins and metabolites for growth in Condition 1, represented here as a ‘blue’ physiology. Likewise, cells may express genes for an ‘orange’ physiology upon sensing Condition 2.

Microbial communities, populations and individuals can each respond to environmental fluctuation. Microbial communities may respond to the introduction of a particular nutrient through a shift in community composition (i.e. increased abundance of microbial members who benefit from that nutrient source) (Fig.   2). In the presence of a particular nutrient, some microbial populations may expand in population size, while less competitive populations decline (Fig. 2). And at the level of single cells, the introduction of a new nutrient may induce distinct gene expression and intracellular metabolite profiles, producing a distinct physiology from when the nutrient was absent (Fig.   2).

**Figure 3.**
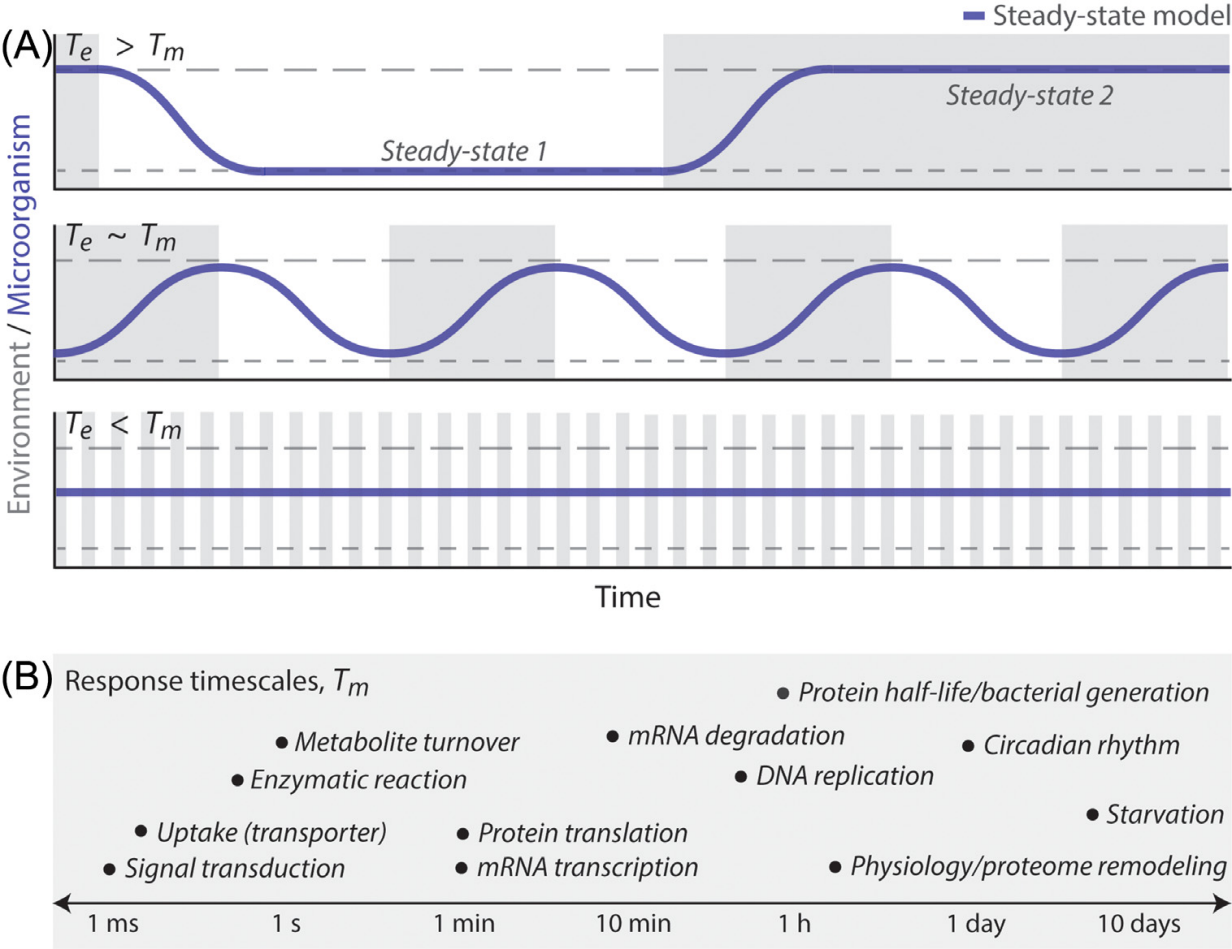
Microbial dynamics depend on the relative timescales between environmental fluctuations and microbial responses. **(A)** The relationship between the timescale of environmental fluctuation (*T_e_*) and the timescale of a microbial response (*T_m_*) determines the dynamics of the response under changing environments. Here, the environment fluctuates between two conditions (gray and white), each associated with a microbial steady state (longer or shorter dashed lines, respectively). When environmental fluctuations occur on sufficiently long timescales relative to the response (*T_e_* > *T_m_*), the response (i.e. the transition between the steady states characteristic of each environmental condition) completes after each shift once *T_m_* has elapsed. When *T_e_* ∼ *T_m_*, the response may produce a behavior that does not stabilize. As *T_e_* becomes smaller with respect to *T_m_*, environmental fluctuations occur faster than the time required to stabilize at steady state, causing microorganisms to fluctuate between steady states without reaching them. Finally, environmental fluctuations can be so fast relative to the microbial response of interest (*T_e_* < *T_m_*) that microorganisms behave as if the environment were a single steady condition. **(B)** Microbial responses to environmental fluctuation span a diversity of behaviors, including changes in nutrient uptake, growth rate, gene expression and more. These responses occur on behavior-specific timescales of milliseconds to days (Shamir *et al*. 2016). In this schematic, we list approximate *T_m_* for a variety of possible responses by order of magnitude, as each process realistically also spans a range. For example, different enzymes will catalyze one reaction at different rates (microseconds to tens of seconds), and bacterial generation times will vary with species and growth condition (tens of minutes to days).

In this review, we demonstrate that the type and timescale of fluctuation can induce responses from microbial communities, populations and individuals, depending on the relative timescales of environmental fluctuation and microbial response (Fig. 3A and B). We lay out a framework that predicts that the response can fluctuate along with the environment, if the environment fluctuates much slower than the full response, such that the response fully completes (i.e. reaches its steady state) before the environment changes once again. This framework also predicts that microbes will not respond to environmental fluctuations, if the fluctuations are much faster than the response timescale (Fig. 3C). Effectively, this framework considers whether microbial responses can reach a steady state in fluctuating environments, possible if the environment fluctuates sufficiently slow or fast relative to the response timescale. We refer to this framework as the steady-state model and, through examples, demonstrate its use as a model for determining the effects of environmental conditions on microbial physiology and ecology.

In the context of the steady-state model, we review studies that directly address the effect of environmental fluctuations on microbial behavior and function, parsing microbial responses by levels of biological organization—from communities, to populations, to individuals. We primarily discuss studies on nutrient fluctuations, but mention selected others to cover a diverse range of fluctuations (e.g. nutrient source, nutrient concentration, oxygen, water availability) and microbial responses. Overall, this review highlights the current knowledge of how fluctuating environments affect microbial behavior to conclude that the observation of a microbial response to environmental fluctuations generates hypotheses for the biological process that may control it. We end by emphasizing important future directions for the study of microbes in nature to carefully consider the temporal fluctuations, experienced by microbial life, that can have major qualitative and quantitative effects for all natural habitats.

## COMMUNITY-LEVEL RESPONSES TO ENVIRONMENTAL FLUCTUATIONS

### The timescale of environmental fluctuations affects community diversity

Studies concerning the role of environmental fluctuations on microbial communities have historically focused on species diversity. The ability of hundreds of microbial species to coexist has long puzzled microbial ecologists (Hutchinson 1961; Pedrós-Alió 2006; Lozupone *et al*. 2012), and understanding how environments can support such diversity remains a fundamental challenge (Chesson 2000). Microscale spatial heterogeneity, resource partitioning, dormancy and environmental fluctuations each provide some explanation for the maintenance of high species diversity. Environmental fluctuations may contribute to high diversity by changing conditions faster than species can go extinct, alternatingly favoring some species over others, but not so fast that the community essentially experiences it as one stable condition (Fig. 3A). This reasoning predicts that, by keeping a microbial community in continuous change, environmental fluctuations may maintain higher species diversity than any single steady environment. In the following examples, we review how experiments in some microbial systems have confirmed this prediction (Rodríguez‐Verdugo, Vulin and Ackermann 2019), whereas others emphasize effects of environmental fluctuation on community metabolism and colonization by new species (Thaiss *et al*. 2014; Kearney *et al*. 2018).

A recent *in vitro* study demonstrated that nutrient fluctuations can maintain a two-species bacterial community when fluctuations occurred faster than the extinction timescales of either species, by measuring community dynamics across alternations between two nutrient sources every 1–5 days (Rodríguez‐Verdugo, Vulin and Ackermann 2019). The first environmental condition provided benzyl alcohol, producing a symbiotic interaction between the two species in which *Acinetobacter johnsonii* metabolized benzyl alcohol and secreted benzoate, the nutrient source for *Pseudomonas putida*. The second condition provided citrate, producing a competitive interaction, as citrate can be directly consumed by both species, that favored *P. putida* in co-culture. In 6-day-long experiments in which the nutrient source fluctuated on 1–3-day timescales, oscillations in community composition maintained both species throughout the experiment, whereas fluctuations on 4–5-day timescales led to the extinction of *A. johnsonii*. These longer fluctuation timescales exceed the timescale required for *A. johnsonii* to go extinct in citrate. In other words, *T_e_* (the nutrient fluctuation timescale) was greater than *T_m_* (the timescale of extinction) (Fig. 3A). Thus, nutrient fluctuations occurring on timescales slower than the timescale on which a species goes extinct will produce lasting change in community composition.

Intriguingly, in the system described above, 1-day fluctuations enabled stable coexistence during the first 40 generations but led to extinctions over evolutionary timescales (∼200 generations), whereas a constant environment could maintain coexistence (Rodríguez-Verdugo and Ackermann 2020). Under daily fluctuations, the abundance of *A. johnsonii* appeared stable for 12 days before decreasing rapidly to extinction 7 days later. Full genome sequencing of single clones from different time points across the long-term experiment found that *P. putida* repeatedly accumulated one or more mutations within 100 generations, mutations allowing the evolved strains to grow at faster rates and to higher yields than the ancestral strain. In contrast, *A. johnsonii* did not acquire mutations over the 100 generations in fluctuating nutrient and was ultimately lost from the community. We will discuss the enhancement of evolution in fluctuating environments in the section on population-level responses. For now, we conclude by emphasizing that whether fluctuations maintain species coexistence over evolutionary timescales appears to depend on the rate at which each member evolves under fluctuations.

### The regularity of fluctuations determines community composition and metabolism

**Figure 4.**
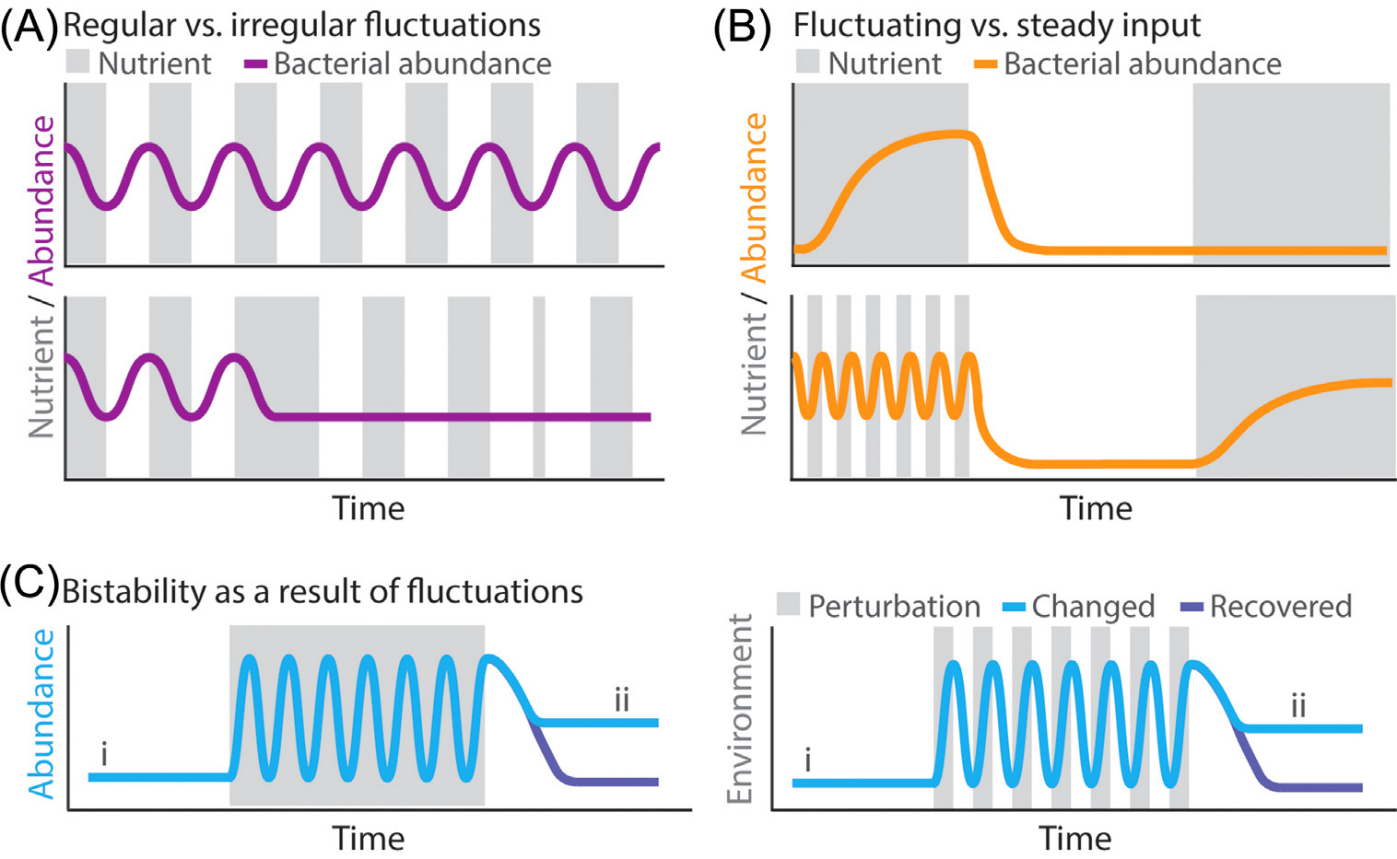
Environmental fluctuations affect the diversity and function of microbial communities. **(A)**The regularity of nutrient fluctuations is essential to produce regular oscillations in the microbial communities of mice (Thaiss *et al*. 2014; Zarrinpar *et al*. 2014) and humans (Thaiss *et al*. 2016). **(B)** Fluctuations in a privileged nutrient source enhance the long-term maintenance of a newly introduced bacterial strain into a pre-existing murine gut microbiota (Kearney *et al*. 2018). The introduced strain could be detected after 60 days without the privileged nutrient if introduced within 35 days of nutrient fluctuations (top), whereas the strain could not be detected when introduced with 35-days of steady nutrient exposure (bottom). **(C)** Environmental fluctuations can induce lasting changes in community composition, even after the environment returns to pre-fluctuation conditions. This bistability (blue) in microbial behavior is observable as a different post-perturbation steady state (ii) than the initial steady state (i). Recent studies have observed such bistability after a single pulse (left, steady gray) (Tropini *et al*. 2018) or series of environmental fluctuations (right, fluctuating gray) (Kearney *et al*. 2018; Nguyen *et al*. 2020).

A similar interaction between timescales may explain how the regularity of environmental fluctuations so substantially impacts the composition and metabolism of microbial communities. Hour-scale nutrient fluctuations have recently been demonstrated to regulate the diurnal rhythmicity of gut microbiome communities. In mice given steady access to food for 24 h a day, the microbiome fluctuated in community composition (measured by 16S rDNA sequencing) and function (i.e. community-level transcription of metabolic pathways) on a 12-h diurnal cycle. Limiting food intake to a 12-h time window within a 24-h period maintained the same diurnal microbiome fluctuations, phase-shifted depending on the time of day food is provided (Thaiss *et al*. 2014). In mice and humans, community composition and the expressed metabolic pathways changed continuously (i.e. did not stabilize) across each 12-h phase (Thaiss *et al*. 2014). Notably, 8–10 h shifts in feeding time—induced in mice by shifting daytime feeding to nighttime feeding and vice versa, and in humans by jetlag—disrupted the regularity of microbiome fluctuations, either inducing erratic fluctuations out of sync with normal daily periodicity or suppressing them entirely (Fig. 4A). While microbiomes can recover their normal periodicity after one-time shifts in feeding time, repetitive disruptions by continued irregularity in nutrient induced long-term disruptions in host metabolism and gene expression commonly found in disease states (Zarrinpar *et al*. 2014; Thaiss *et al*. 2016).

Altogether, the responses from these complex intestinal communities suggest that nutrient fluctuations regulate gut community metabolism by fluctuating on a specific timescale. Perturbing this timescale by either lessening or increasing the time between meals altered the homeostasis of community composition and metabolism, likely because different feedings time introduce new nutrient to different states of the intestinal microbial community. Thus, the timescale of environmental fluctuation not only affects community composition by limiting microbial extinction, but also serves a homeostatic function in regulating the extent to which community metabolism affects the environment and in turn, community composition.

### Resource fluctuations can enhance microbial colonization

Environmental fluctuations can serve another key role in controlling community composition by promoting the colonization of a new species. After exposing mice to the desired transplant *Bacteriodes plebeius*, a recent study compared the maintenance of *B. plebeius* in gut microbiomes exposed to 35 days of either fluctuating or steady provisions of seaweed, a ‘privileged’ nutrient source that only the introduced *B. plebeius* strain could metabolize. Sixty days after the last exposure to seaweed, *B. plebeius* could still be detected by quantitative PCR in the gut community of mice previously fed a diet of fluctuating seaweed (4 days with 1% seaweed, 4 days with 0%). In contrast, *B. plebeius* was undetectable in the community of mice previously fed a constant 1% seaweed diet, despite the fact that this diet supplied a greater overall amount of seaweed (Kearney *et al*. 2018). Thus, compared with a steady supply of seaweed, fluctuating seaweed availability enabled the incorporation of *B. plebeius* into a pre-existing stable gut microbiome. The fluctuating seaweed diet produced a novel, stable community composition, which did not return to the prior steady-state community composition of a mouse never fed seaweed even after seaweed was no longer available (Fig. 4B).

Precisely how fluctuating environmental conditions induce new steady-state community compositions remains to be explained, but the timescale of fluctuations that elicit a response (i.e. colonization) may provide an important clue. It is not that the duration of seaweed availability was insufficiently long for the colonization process to occur. Instead, the 35-day duration of the steady seaweed exposure may be sufficiently long for another process—one inhibitory to colonization—to occur: a process that does not occur with repeated 4-day exposures and appears to involve interactions between *B. plebeius* and the surrounding environment (e.g. neighboring community members and the host immune system) (Fig. 4C). In mice, *B. plebeius* cells can be bound by the host-secreted antibody, IgA (Kearney *et al*. 2018), which controls host-mediated retention (Donaldson *et al*. 2018) and expulsion of bacteria in a growth rate dependent manner (Hoces *et al*. 2020). Thus, fluctuating seaweed may benefit *B. plebeius* growth enough to provide a niche for colonization, while maintaining a slower growth rate than steady seaweed, preventing IgA-mediated expulsion of fast-growing bacteria from the gut (Hoces *et al*. 2020). Indeed, a separate study found that *S. typhimurium* maintains a steady abundance in mouse guts until 4 days of fast growth, after which it is increasingly expelled in an IgA-dependent manner (Moor *et al*. 2017).

### The relative timescales of environmental and community function

We finalize this section on microbial communities with a more detailed discussion on the how environmental fluctuations can affect community function. Like intestinal communities (Thaiss *et al*. 2014), the metatranscriptome of marine microbial communities can also fluctuate with the environment. One study monitored transcriptional activity from 1-L samples of the ocean's surface, sampling periodically over 2 days with a sampler that drifted in the water along with the community, and found coordinated fluctuations in gene expression across the bacterial community (Ottesen *et al*. 2013). The gene expression profiles of photosynthetic microbes closely followed diel cycles and included genes controlled by circadian rhythms, which we discuss further in the single-cell section. This transcriptional rhythm was recently found to be synchronized with pigment abundances (Becker *et al*. 2020). Here, we emphasize that this rhythm was synchronized across diverse phytoplankton, demonstrating the strong influence exerted by the 24-h light cycle over photosynthetic communities.

The fluctuations of heterotrophic bacteria, observed in growth-related genes (i.e. ribosomal and oxidative phosphorylation), were also synchronized across diverse species yet did not occur on a 24-h cycle, suggesting that heterotrophic responses were not controlled by the rhythms of the primary producers (Ottesen *et al*. 2013). Instead, the synchronous hour-scale fluctuations in heterotrophic transcription may reflect a coordination of community function to stochastic environmental fluctuations. Because gene transcription occurs on the timescale of 1 min and mRNA degradation occurs on the timescale of 10 min (Shamir *et al*. 2016), hour-scale transcriptome fluctuations are highly suggestive of hour-scale environmental fluctuations. The source of these environmental fluctuations is currently unknown. However, by observing the temporal dynamics of marine metatranscriptomes, the knowledge that some as-yet unknown process induces hour-scale fluctuations in heterotrophic growth-related gene expression enables future studies to target its identity and source.

Some bacterial communities, however, do not fluctuate in gene expression despite the presence of strong environmental fluctuations. In a recent study on coastal sediments, microbial communities were found to be continuously able to denitrify even as the concentration of oxygen—considered a denitrification inhibitor—fluctuated on hour timescales (Marchant *et al*. 2017). Specifically, the total abundance of denitrification transcripts was unchanged by fluctuations in oxygen, suggesting that these coastal sediment communities have evolved alternative mechanisms of regulating denitrification pathways such that they are not responsive to oxygen fluctuations. That insensitivity to oxygen was not previously observed in the expression of denitrification points toward community-specific adaptations to fluctuations that, in this case, may increase a community's capacity to denitrify amidst changing environments.

The implication of environmental fluctuations as a selective pressure has also been borne out of observations from the alternating oxic and anoxic conditions experienced by some soil microbial communities, which simultaneously perform CO_2_ respiration, methanogenesis, N_2_O production and iron reduction, as observed through direct chemical measurements (DeAngelis *et al*. 2010). More taxa from these multi-tasking communities were found to be more actively growing (i.e. higher ribosomal RNA to DNA ratios) when exposed to 4-day fluctuations in redox state than in static oxic or anoxic conditions, suggesting that members of these soil communities are adapted for growth in fluctuating rather than static redox conditions (DeAngelis *et al*. 2010).

These last two studies (DeAngelis *et al*. 2010; Marchant *et al*. 2017) are suggestive of an evolutionary response that enables bacteria to maintain metabolic rates despite strong persistent fluctuations in the environment. While the oxygen fluctuations in these last two studies were macroscopically observed over hours or days, the microscopic (pore-scale) manifestation of these fluctuations in soils and sediments could occur as fast as minutes. Certain adaptations, such as a lack of transcriptional sensitivity to oxygen concentration, may be specifically beneficial in environments that fluctuate rapidly by enhancing microbial metabolism and overall community function.

## POPULATION-LEVEL RESPONSES TO ENVIRONMENTAL FLUCTUATIONS

### Environmental fluctuations can promote evolution and diversity

**Figure 5.**
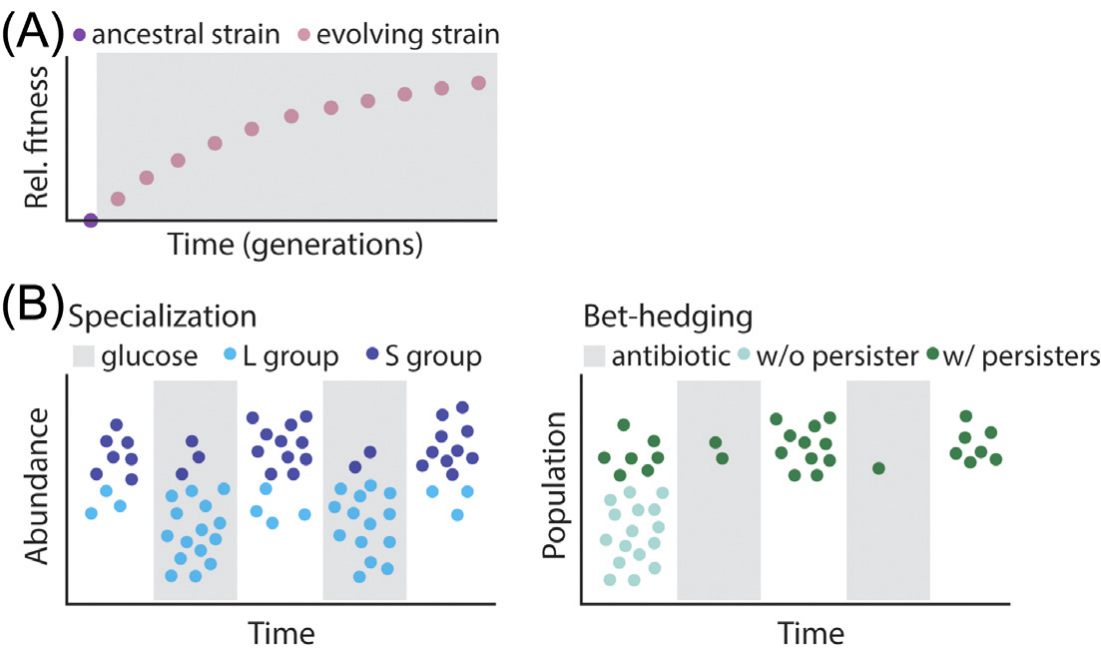
Environmental fluctuations drive the evolution of adaptive traits in microbial populations. **(A)** Environmental change drives evolutionary adaptations. Experiments propagating *E. coli* in a constant environment have shown that the fastest rates of evolution adaption occur within the first 5000 generations evolved in a new environment (gray), after which the accumulation of beneficial mutations begins to slow (Cooper and Lenksi 2000; Tenaillon *et al*. 2016). **(B)** Evolutionary adaptations in fluctuating environments can be classified into two groups: (i) specializations and (ii) bet-hedging adaptations. Specializations are adaptations that increase population growth in one of the multiple conditions that the environment fluctuates between, such as the L group's advantage in glucose and the S group's advantage in zero glucose (Rozen and Lenksi 2000; Rozen *et al*. 2009). Bet-hedging adaptations, such as persister cells in *E. coli* populations (Balaban *et al*. 2004), decrease population growth in any one condition but increase population growth when the population experiences fluctuations. Colors specify populations (i.e. populations with or without persisters), not phenotypes (i.e. persister vs normal growing cell).

Populations are the fundamental unit of evolution. Thus, environmental fluctuations affect not only population growth and size but also the emergence and spread of new traits. Indeed, evolution experiments with microorganisms have found that genetic adaptation may be especially rapid when microbial populations are exposed to environmental fluctuations (Elena and Lenski 2003). In these experiments, an ‘ancestral’ strain is evolved in a new environment for tens of thousands of generations. This evolved strain is then mixed in a 1:1 ratio with the unevolved ancestral strain, and the emergent relative abundance of evolved cells to ancestral cells after 1 day of competition is called the evolved strain's relative ‘fitness’ in the new environment. From 12 evolved *Escherichia coli* populations, average gains in population fitness were 10-fold greater between the first 5000 generations of evolution than between the 15 000th and 20 000th generations (Cooper and Lenksi 2000). The fraction of mutations that are beneficial decreases with rising fitness (Tenaillon *et al*. 2016), indicating that genetic adaptations to new environments are initially rapid and slow over time (Fig. 5A). Many environmental fluctuations occur on timescales much shorter than 5000 microbial generations, and thus fluctuations can create environments in which microorganisms rapidly evolve.

In addition to accelerating evolution, environmental fluctuations may lead to greater genetic variation among microbial populations. Populations evolved for 2000 generations in fluctuating nutrient environments (e.g. daily alternations between glucose and lactose) acquired significantly different adaptations between replicate populations (i.e. same ancestral strain and fluctuating environment), whereas replicate populations evolved in the same steady nutrient environments (e.g. only glucose or only lactose or both glucose and lactose) did not differ in their evolved adaptations (Cooper and Lenksi 2010). The greater variability between populations evolved in fluctuating environments highlights the diversity of adaptations that can evolve in response to environmental fluctuations.

### Specialist adaptations maintain microbial diversity in fluctuating environments

Some adaptations acquired in fluctuating environments enhance population growth within one of the multiple conditions that an environment may fluctuate between. For example, one study evolved *E. coli* for ∼20,000 generations in daily fluctuations between glucose-available and glucose-deficient conditions, observing the diversification of two genotypes. One population (L) had a growth advantage when glucose was available; the other population (S) could grow after the glucose was depleted, whereas the L population could not (Rozen and Lenksi 2000; Rozen *et al*. 2009). The stable coexistence of these populations, enabled by the frequency of glucose fluctuations, was quantified by plating and exemplifies how environmental fluctuations can induce genetic adaptations and maintain diverse genotypes when they occur on timescales that prevent extinction (Fig. 5B).

Environmental fluctuations may also maintain diverse genotypes in a population by affecting the density dependence of traits. Fluctuations in the physical, chemical or biological components of the environment create variation in selective pressures, ultimately introducing fluctuations in the population over time (Saether and Engen 2015). Some traits tend to have higher growth rates when population density is high. For example, a large population of ‘public good’ producers will tend to favor the growth of ‘non-producers’, cells that exploit the public good yet do not contribute to its production (Chuang, Rivoire and Leibler 2009; Ross-Gillespie *et al*. 2009). However, despite the faster growth rate of non-producers, producers may be maintained in the population via a variety of fluctuations. One study found that fluctuations that create periodic bottlenecks in an *E. coli* system of producers and non-producers enabled an overall increase in producers (Chuang, Rivoire and Leibler 2009). Another study found that population structure determined whether non-producers even had a growth advantage over producers. Only when in close proximity to producers (in this case, cells that secreted siderophores, i.e. iron scavenging proteins) did non-producers have increased growth rates with higher population densities (Ross-Gillespie *et al*. 2009). Thus, environmental fluctuations that affect the population size or structure may determine how seemingly disadvantaged traits may persist in a population.

### Fluctuation timescale can control the fixation of new traits

Whether a novel adaptation takes over a population or co-exists with unevolved variants can depend on the timescale of environmental fluctuations. A recent study engineered an *E. coli* strain to stochastically switch from tetracycline (Tc) susceptible to Tc resistant, mimicking the spontaneous occurrence of antibiotic resistance (Lin and Kussell 2016). To study the fixation of resistance in populations under fluctuating antibiotic, clonal populations of this strain were exposed to fluctuations of Tc, in which Tc concentrations periodically shifted between killing (i.e. higher than the minimum inhibitory concentration) and recovery (i.e. no antibiotic) at timescales ranging from 6 min to 6 h. The Tc timescale determined what fraction of each population, initially composed of 95% susceptible cells, consisted of resistant cells after 48 h of fluctuations. On average, between 10% and 20% of each population exposed to fluctuations on timescales between 15 min and 6 h was replaced by resistant cells, whereas resistant cells became nearly 80% of each population exposed to 6 min fluctuations. In other words, when the timescale of antibiotic fluctuation was sufficiently shorter than the timescale required for susceptible cells to maintain a growth advantage (*T_e_* < *T_m_*), cells effectively experienced a mixed Tc concentration such that resistant cells had a growth advantage. Intermediate timescales (*T_e_* ∼ *T_m_*) enabled the maintenance of both phenotypes in the population (Fig. 3A), as the killing and recovery phases were sufficiently long for the fluctuating environment to alternatingly favor one phenotype with each environmental condition.

### Bet-hedging as an adaptation to unpredictable fluctuations

Some microorganisms have evolved traits that do not to specialize in a single condition, but improve survival in environments that fluctuate between multiple conditions. *De novo* evolution of adaptations specifically to enhance growth in fluctuating environments has been observed through experimental evolution. One study observed the evolution of stochastic state switching in populations of *Pseudomonas fluorescens* evolved in a fluctuating environment that continually favored new phenotypic states (Beaumont *et al*. 2009). Bet-hedging, or the stochastic production of distinct phenotypic states, is perhaps the best-known case of a population-level adaptation for survival in fluctuating environments. For example, populations of genetically identical *E. coli* produce both normal growing cells and non-growing persister cells, which make up 0.0001% of a fast-growing population (Balaban *et al*. 2004). Bet-hedging strategies like the production of persisters are considered an insurance policy against sudden environmental change, for example enabling at least some members of a population to survive an antibiotic treatment and re-seed a population after conditions once again permit growth (Fig.   5B). The persister phenotype is non-heritable and is instead spontaneously induced, so that populations derived from single persister cells maintain the same frequency of persisters as populations that have not experienced an antibiotic challenge (Balaban *et al*. 2004).

The proportion of persisters in a population may reflect the frequency of environmental fluctuations. The low wild-type abundance of persisters suggests that the ratio of persisters to normal cells is well suited for environments in which antibiotic stress is a rare event, whereas environments with frequent antibiotic exposure have been predicted to favor mutant cells with 1000-fold higher proportions of persisters (Wolfson *et al*. 1990; Kussell *et al*. 2005). A recent study engineered two populations of yeast to switch between two growth phenotypes stochastically, without environmental input, with one population switching at higher rates than the other (Acar, Mettetal and Van Oudenaarden 2008). These populations were subjected to nutrient fluctuations between two environments: the first environment favoring one phenotypic state and the second environment favoring the other. When exposed to various frequencies of nutrient fluctuations, fast-switching populations outgrow slow-switching populations when the environment fluctuates rapidly, and slow-switching populations outgrow fast-switching populations when the environment fluctuates slowly (Acar, Mettetal and Van Oudenaarden 2008). Thus, the proportion of different phenotypic states is likely a tunable trait for populations evolving under fluctuating environments.

The production of distinct states, or phenotypic heterogeneity, is employed by diverse microorganisms to hedge their bets against environmental change. Fast-growing clonal populations of pathogenic *Salmonella typhimurium* contain two subpopulations—one that expresses the flagellar protein FliC and one that does not—enabling the population to benefit from motility and chemotaxis in certain compartments within a host, while evading the heightened anti-flagellar immune responses in others (Stewart *et al*. 2011). Population-level heterogeneity in *E. coli* metabolism has also been suggested as a bet-hedging strategy to protect a population's ability to grow when the nutrient source fluctuates. Upon a shift from glycolytic (i.e. environment provides glucose) to gluconeogenic (i.e. environment provides a non-carbohydrate carbon source such as acetate) nutrient conditions, a phenotypically uniform population splits into two subpopulations: one that physiologically adapts to grow on the gluconeogenic nutrient source and one that ceases growth until glucose returns to the surrounding environment (Kotte *et al*. 2014). Similarly, the concentration of ammonium, the substrate preferred over N_2_ by N_2_-fixing *Klebsiella oxytoca*, determines the rate of N_2_ fixation in the presence of ammonium, by regulating the fraction of individuals consuming N_2_ (Schreiber *et al*. 2016). Lower external ammonium concentrations induced larger subpopulations of N_2_-fixing cells, providing a growth advantage to populations in environments that can be suddenly deplete in ammonium concentration. Finally, yeast respond to environmental fluctuations by leveraging molecular noise (i.e. stochastic differences in gene expression within individuals of a population) to protect the population from unpredictable conditions. When the environment changes abruptly, yeast trigger general stress responses with noisy gene expression relative to that of growth-related genes (López-Maury, Marguerat and Bähler 2008).

Some bet-hedging strategies employ heritable adaptations and occur on evolutionary timescales. Populations of opportunistic pathogens experience fluctuating exposure to host immune responses and hedge their bets by changing not their gene expression but their genotypes (Rainey *et al*. 2011). For example, *Haemophilus influenza* is a normal member of the microbiota in healthy human respiratory tracts, yet can cause disease (e.g. meningitis, pneumonia) when the opportunity arises (Moxon, Bayliss and Hood 2006). To maintain pathogenicity while evading the immune system, *H. influenza* and other bacterial pathogens reversibly switch between genotypes at relatively high frequencies. Hypermutability at DNA sequences (specifically, those associated with cell surface proteins) generates populations with diverse surface structures—some recognized as pathogenic antigens, some not—enabling the bacterial population to prepare for rapid and unpredictable environmental fluctuations (Moxon *et al*. 1994; Moxon, Bayliss and Hood 2006). Microbial evolution has also been demonstrated to drive evolutionary bet-hedging in multicellular organisms. Experiments with the nematode *C. elegans*, which can reproduce sexually and asexually, have found that coevolution with a bacterial parasite (*Serratia marcescens*) leads to populations of *C. elegans* with significantly more outcrossing (Morran *et al*. 2011). Populations of *C. elegans* that reproduced only asexually exhibited higher mortality rates when exposed to *S. marcescens* strains that had been coevolved in *C. elegans* compared with populations that reproduced sexually, supporting the concept that increased genetic (and therefore phenotypic) variability provides an advantage in ever changing environments.

Overall, by producing phenotypic heterogeneity, even a genetically clonal population can increase the likelihood that at least some individuals of the population will survive or even perform well under fluctuating environments, particularly unpredictable ones (Veening, Smits and Kuipers 2008a; Rainey *et al*. 2011; Ackermann 2015).

### Diverse effects of fluctuations on phenotypic heterogeneity

**Figure 6.**
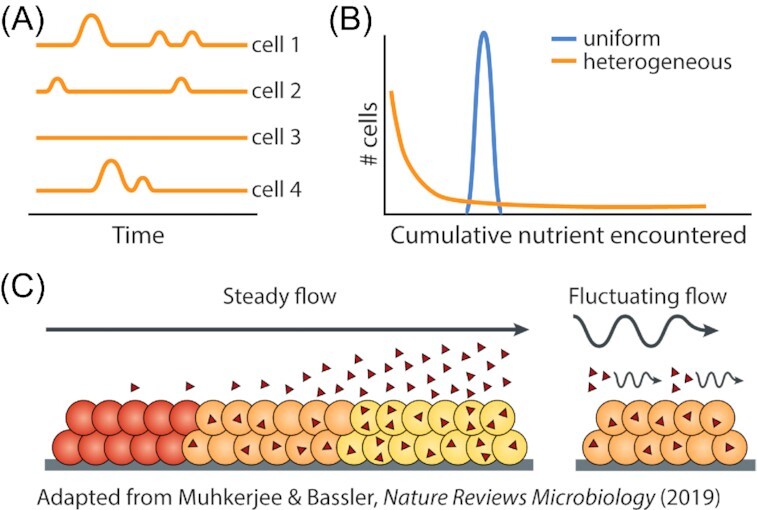
Environmental fluctuations affect the phenotypic heterogeneity of microbial populations. **(A)** Variation in temporal exposure to nutrient between individual cells of a clonal population arises due to the heterogeneous spatial structure of microbial habitats. Individuals located in different regions of a heterogeneous habitat encounter different nutrient patches at different times, leading to heterogeneity in nutrient encounter and cellular physiology between cells. **(B)** Distributions of nutrient encounter by individuals within a microbial population in uniform and heterogeneous environments. Because individuals in heterogeneous environments experience differing times and amounts of nutrient exposure (Kuzyakov and Blagodatskaya 2015), the vast majority of cells do not encounter enough nutrient to divide. Thus, the temporal fluctuations in resource concentration (panel A) produced by environmental spatial heterogeneity can increase the phenotypic heterogeneity within microbial populations. **(C)** Fluctuations in fluid flow can reduce population heterogeneity by affecting the accumulation of cell-to-cell signaling molecules (Muhkerjee and Bassler 2019). Under steady fluid flow, quorum sensing molecules (red triangles) accumulate to higher concentrations around regions of the bacterial population that are further downstream. This gradient in quorum sensing molecules produces a gradient in gene expression and therefore microbial activity within a population. Such gradients are lessened when fluid flow fluctuates between flowing and stagnant, enabling a more homogeneous expression of quorum sensing-mediated microbial functions.

Environmental fluctuations can also produce phenotypic variability within populations, not only through bet-hedging mechanisms, but also via encounter rate distributions between individuals residing in patchy resource landscapes (Fig. 6A and B). Clonal individuals of free swimming organisms can encounter vastly different amounts of nutrient, such that only a small percentage of the population accumulates enough nutrient to divide (Coronado, Valtat and van der Meer 2015). This heterogeneous distribution of nutrient among a population arises from the spatial heterogeneity in how bacteria and nutrient are distributed and can be further accentuated by heterogeneities in cell shape and fluid flow (Słomka *et al*. 2020). Thus, the heterogeneous distributions of environmental factors produce striking differences in the temporal environment experienced between individuals within a microbial population.

Finally, environmental fluctuations may also reduce phenotypic heterogeneity within a population, particularly when fluctuations occur on fast timescales. While stochastic heterogeneity within a clonal population arises in bacterial quorum sensing (Boedicker, Vincent and Ismagilov 2009; Cárcamo-Oyarce *et al*. 2015), this phenotypic heterogeneity can be modulated by the local cell density and environmental conditions (Fig. 6C). Quorum sensing is a collective behavior by which cells sense population density and control the expression of density-dependent behaviors such as virulence factor production or biofilm formation. It has been suggested that fluctuations in fluid flow (i.e. alternations between high and low flow rates) can produce fluctuations in bacterial quorum sensing (Muhkerjee and Bassler 2019). Fluctuations in fluid flow alter the accumulation of secreted quorum sensing molecules (autoinducers), which synchronize the gene expression of collective behaviors (e.g. DNA uptake) across a bacterial population. Because fluid flow removes autoinducers from the environment around the cells, populations in steady flow conditions experience strong gradients in autoinducer concentration, as downstream members of the population accumulate higher autoinducer concentrations than those upstream (Kim *et al*. 2016). This autoinducer gradient is likely weakened when flow fluctuates (Muhkerjee and Bassler 2019), as phases of no flow allow autoinducer molecules to accumulate (e.g. throughout the biofilm), with faster fluctuations in flow potentially leading to more uniform gene expression than that of a population in a fluid flow rate that is slower to change.

In summary, environmental fluctuations affect the evolution and heterogeneity of microbial populations: increasing heterogeneity in many cases, potentially decreasing heterogeneity in others. The exact response of the population depends on the timescale of fluctuations and how predictable they are. In general, slow enough fluctuations allow evolution to optimize a population for growth in a specific condition, conferring the population a growth advantage when that environmental condition is present. Such may be the case when distinct populations of the same species co-exist within an environment (i.e. found in the same gut) (Arevalo *et al*. 2019). Sporadic enough fluctuations confer an advantage to a population to hedge its bets across diverse environmental conditions, ensuring that some members of the population survive as the environment fluctuates.

Overall, the diverse strategies employed by microbial populations to respond to these fluctuations emphasize that environmental fluctuations can represent a selective pressure to which microorganisms evolve. We discuss in the next section one final group of adaptations: adaptations that also confer benefits in fluctuating environments (rather than one environmental condition), but operate at the level of single cells.

## SINGLE-CELL LEVEL RESPONSES TO ENVIRONMENTAL FLUCTUATIONS

It has been long understood that microbes adapt their growth and physiology to their environment. Currently, our understanding of single-cell responses to environmental change derives heavily from characterizations of single physiological transitions from one steady state to another. Thus, the steady-state model is the prevailing framework by which single-cell physiology in dynamic environments is considered. Under this model, a physiological steady state (e.g. growth rate, size or proteome) exists for each environmental condition (Schaechter, Maaløe and Kjeldgaard 1958; Scott *et al*. 2010). Upon sensing a shift in environmental conditions, cells initiate a cascade of responses to reach the physiological steady state characteristic of the new condition (Kjeldgaard, Maaløe and Schaechter 1958; Leveau *et al*. 1999; Erickson *et al*. 2017). However, what happens when a cell experiences environmental fluctuations on timescales faster than the timescale required to fully reach the corresponding physiological steady states? Recent studies of microbial growth under fluctuating conditions have observed responses that are independent of this latter timescale, finding a different response to repeated fluctuations than single environmental shifts. These responses again demonstrate how microbes have evolved specific adaptations for fluctuating environments including at the level of single cells, which represents the focus of this section.

### Microbial anticipation of environmental fluctuations

Some microorganisms have evolved the ability to anticipate environmental fluctuations and initiate behaviors prior to a change in environmental conditions, rather than after. Temporally regulated gene expression is one means by which microbes anticipate a change and physiologically prepare before the change occurs. Like plants and animals, fungi and cyanobacteria use endogenous circadian rhythms to coordinate their metabolism with daily cycles of sunlight, anticipating environmental changes (e.g. sunrise) and initiating changes in mRNA and protein abundance in advance (Cohen and Golden 2015). For some cyanobacteria, this cycling of gene expression temporally separates photosynthesis from nitrogen fixation: two processes that are inefficient to co-express within the same individual, given that oxygen (a product of photosynthesis) inhibits nitrogenase activity (Golden *et al*. 1997). Instead, single cells manage both metabolic processes by photosynthesizing during the day and upregulating nitrogenase activity just before the onset of darkness. These diel fluctuations in gene expression are genome-wide and regulated, at least in the cyanobacterium *Synechococcus elongatus*, by the clock gene cluster *kaiABC*, likely through fluctuations in the phosphorylation state of the transcription factor KaiC (Ishiura *et al*. 1998). This circadian rhythm produces daily oscillations in mRNA and protein even when cyanobacteria are transferred into an environment with continuous light, demonstrating how the periodicity of expression is an evolved anticipatory response rather than a response triggered by an environmental cue (Golden *et al*. 1997).

Some environmental fluctuations are not as periodic as day–night cycles yet predictable enough for microorganisms to evolve adaptations to anticipate them. For example, *Actinobacteria* are heterotrophs that have no photosystems; they cannot convert light into chemical energy. Yet, *Actinobacteria* grow faster and transcriptionally upregulate sugar transport and breakdown pathways in light (Maresca *et al*. 2019). This upregulation of cellular activity in light is thought to be a means by which *Actinobacteria* anticipate the release of organic material from primary producers and adjust their physiology to prepare for the upcoming increase in nutrient availability.

**Figure 7.**
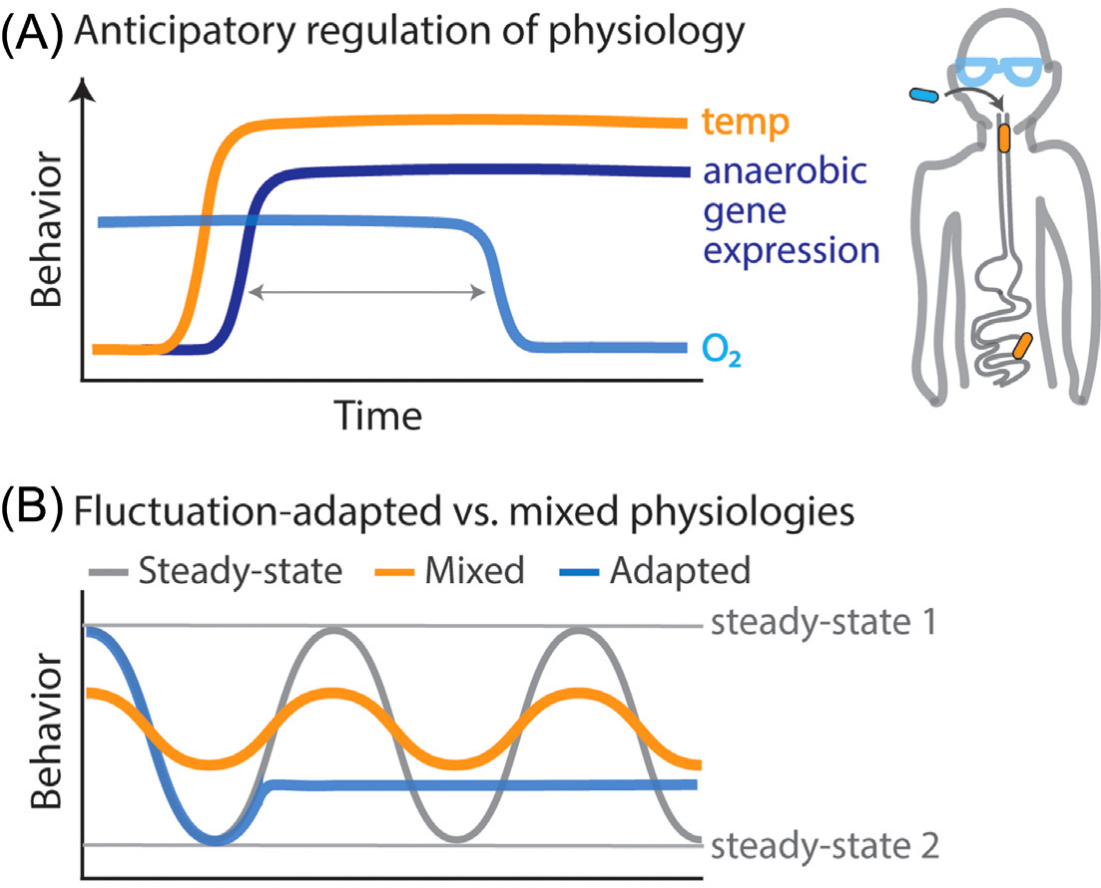
Physiological adaptations to environmental fluctuations at the single-cell level. **(A)** Some microorganisms anticipate environmental change by sensing the occurrence of an environmental condition that reliably precedes the anticipated condition. Increased temperature is one such example. *E*.*coli* upregulates genes for anoxic conditions long before experiencing a decrease in oxygen concentration (Tagkopoulus, Liu and Tavazoie 2008). **(B)** Some single-cell physiologies that emerge in fluctuating environments are distinct from steady-state physiologies. Some may arise while following the steady-state model, if the environment fluctuates on timescales faster than cells can reach each physiological steady state. This produces mixed physiologies, with partial expression of genes associated with both steady states. Alternatively, some single-cell physiologies are novel fluctuation-adapted physiologies, induced by environmental fluctuations to provide a growth advantage when environments fluctuate at certain timescales.

Anticipatory adaptions also enable *E. coli* to physiologically prepare for environmental fluctuations in the gut. As it travels along the mammalian gut, *E. coli* encounters lactose before maltose and thus activates the expression of maltose operons upon exposure to lactose. This transcriptional coupling was lost in laboratory-evolved strains grown in lactose without subsequent exposure to maltose for 500 generations (Mitchell *et al*. 2009). Similarly, upon entering the oral cavity of the mammalian host, *E. coli* uses the increase in temperature as a predictive signal for the impending drop in O_2_ levels characterizing the near anaerobic gut (Tagkopoulus, Liu and Tavazoie 2008) (Fig. 7A). In wild-type cells, increased temperature triggers the transcription of anaerobic pathways prior to the environmental drop in oxygen. Experiments evolving *E. coli* in an environment that fluctuated every 40 min between high temperature with high oxygen and low temperature with low oxygen—both conditions opposite to the wild-type association—successfully decoupled the sensing of temperature from oxygen-related physiological regulations. Strains evolved after 100 h of evolution in the fluctuating environment exhibited reduced transcription of anaerobic genes after a temperature shift when compared with the ancestral strain (Tagkopoulus, Liu and Tavazoie 2008). The evolution of this decoupling suggests that transcriptionally mediated anticipatory adaptations are advantageous in predictable environmental fluctuations slower than the timescale required for cells to physiologically remodel (*T_e_* > *T_m_*). Temperature and oxygen fluctuations occurring much faster than protein turnover timescales would cause cells to be maladapted to frequently occurring environmental conditions, by containing proteins specific to opposite environments.

In summary, diverse microorganisms have evolved anticipatory adaptations that physiologically prepare a cell for life in a specific condition (e.g. anaerobic metabolism) before that condition is present (e.g. low oxygen). These adaptations temporally regulate gene expression to prepare cells for predictable environmental fluctuations occurring on timescales substantially slower than that required to physiologically remodel (i.e. fully adjust the proteome). We next discuss possible single-cell adaptations for growth in environments than fluctuate substantially faster.

### Stable physiologies despite environmental fluctuations

Single bacterial cells appear to have physiological adaptations that benefit growth in minute-scale nutrient fluctuations. A recent study exposed *E. coli* to two different environments: (i) a fluctuating environment in which nutrient fluctuated between a high and low concentration on timescales between 30 s to 60 min and (ii) a steady environment (e.g. high nutrient) in which cells reach steady-state growth before experiencing a nutrient shift to the other steady environment (e.g. low nutrient) (Nguyen *et al*. 2020). Quantifications of single-cell growth rate from each environment, which used the same high and low nutrient concentrations, revealed distinct growth rate dynamics between cells grown in fluctuating vs. steady environments. After a nutrient shift, cells grown in a steady environment transitioned to a new steady-state growth rate over a timescale of hours. In contrast, cells grown in fluctuating environments showed an extremely rapid response, stabilizing in growth rate within minutes after a nutrient shift, but at a growth rate lower than the characteristic steady-state growth rate of the immediate nutrient concentration. This stability in growth rate indicates that these cells had adopted a stable fluctuation-adapted physiology, rather than continuously transitioning between the steady-state physiologies characteristic of high and low nutrient (Fig.   7B). This deviation in growth rate dynamics between steady and fluctuating environments demonstrates how stable bacterial physiologies can emerge after exposure to environmental fluctuations occurring on minute timescales.

Physiological stability despite environmental change can confer growth or motility advantages during environmental fluctuations, particularly those on fast timescales. *E. coli* physiologically adapted to minute-scale nutrient fluctuations exhibited a greater average growth rate than predicted based on the growth rate dynamics of steady-state-adapted cells exposed to single shifts in nutrient concentration (Nguyen *et al*. 2020). The molecular underpinnings of this physiology remain to be elucidated, though it appears that fluctuation-adapted cells suspend transitions between steady-states in favor of prioritizing higher immediate growth rates. A stability or unresponsiveness to environmental change, or noise, has been similarly theorized to increase growth rate in microbial populations exposed to relatively quickly fluctuating environments (Melbinger and Vergassola 2015).

The expression of bacterial motility has also been observed to be stable despite environmental fluctuations in hydration. *P. putida* requires an aqueous environment to swim; however, after a period of dry conditions, motility rapidly resumes upon the return of hydrated conditions. One study exposed wild-type *P. putida* and flagellar mutants (*∆fliC*) to overwhelmingly dry conditions, punctuated by increases in water availability for 5 min at a time, twice per day, and found that wild-type cells exhibited greater dispersal than mutants, which exhibited comparable dispersal to wild-type cells exposed to only dry environments (i.e. no pulse in water availability) (Dechesne *et al*. 2010). The ability of wild-type cells to disperse within such fleeting exposures to water indicates that the ability to swim was stably maintained throughout the dry periods and activated immediately upon the replenishment of water. Thus, it appears that the rapid and stochastic nature of some environmental fluctuations may necessitate the stability of certain physiological functions (i.e. growth and motility) in order for those functions to be an advantage to individual cells.

### Timescale-dependent effects on microbial growth and gene expression

Which environmental timescales produce the most accentuated effects on microbial physiology depends on the timescale of key physiological functions within the cell. *E. coli* do not simply average their nutrient environment, even when fluctuations are on timescales as fast as seconds. When nutrient fluctuated between 30 s to 60 min timescales, the average growth rate of individual *E. coli* cells was observed to decrease with increasing nutrient timescales (Nguyen *et al*. 2020). For nutrient fluctuations much slower than 60 min, growth rate was predicted to increase with increasing nutrient timescale. A similar study measured the growth rate of photosynthetic microorganisms, the cyanobacteria *Synechococcus elongatus* and the alga *Chlamydomonas reinhardtii*, under fluctuating light conditions on timescales ranging between 0.1 s and 27 h and found that fluctuations occurring every 10–100 s resulted in the greatest losses in growth rate (Graham *et al*. 2017). These 10–100 s timescales, *T_e_*, are on the order of the slow deactivation kinetics of Rubisco Activase, *T_m_*, which activates in light and deactivates without irradiance with a lag time that substantially penalizes growth at these timescales (*T_e_* ∼ *T_m_*). Fluctuations in light at faster or slower timescales suffered less of a growth penalty, by escaping the lag time penalty (*T_e_* < *T_m_*) or diminishing it (*T_e_* > *T_m_*). This form of timescale-dependency in growth due to protein-specific kinetics likely applies not only to enzymes, like Rubisco Activase, but also transporters and all other molecules with process-determining biochemical kinetics.

Environmental fluctuations on timescales similar enough to protein expression timescales can produce mixed physiologies within individual cells and reduce lag times generally associated with microbial responses. Because glucose represses the expression of lac operon genes (i.e. genes for growth on lactose) (Görke and Stülke 2008), *E. coli* previously grown in steady glucose upon the start of fluctuations respond to the first shift to lactose with a lag time in growth (Lambert and Kussell 2014). Growth rate drops immediately after the shift to lactose before increasing again, as proteins for growth on lactose are synthesized. However, *E. coli* subjected to minute-scale fluctuations between glucose and lactose have been shown to exhibit little to no lag in growth rate, as measured by imaging a population's single-cell elongation rate, suggesting that a mixed physiology in which cells contain protein for growth on both can arise in response to fluctuating environments (Lambert and Kussell 2014). Minute timescales are long enough to enable mRNA transcription and protein translation, yet shorter than protein half-lives (Fig. 3B). Thus, under minute-scale fluctuations between glucose and lactose, lac operon proteins produced during exposures to lactose can persist through the glucose phases to enable growth on lactose when it returns. This poor synchronization of transcriptional activity and protein abundance is a feature of microbial gene expression in dynamic environments (Moran *et al*. 2013) and represents another mechanism by which microbial physiology is controlled by environmental fluctuations (Fig. 7B).

This form of protein memory, accrued over repeated fluctuations, may provide individual cells a means of sensing and responding to the timescale of environmental fluctuations. A recent study proposed a model by which the accumulation of FtsZ, a bacterial cell division protein, can link the timing of nutrient fluctuations with the timing of cell division (Sekar *et al*. 2018). Experimentally, *E. coli* were starved in carbon-free environments until they ceased to divide before then being exposed to intermittent 10 s pulses of glucose every 3–5 min. Upon exposure to glucose fluctuations, cells exposed to higher frequencies (3 min) resumed cell division faster than cells exposed to lower frequencies (5 min). In the model, extracellular glucose fluctuations drove intracellular FtsZ fluctuations, which induce cell division once the cell accumulates a threshold concentration of FtsZ (Sekar *et al*. 2018; Si *et al*. 2019). Thus, the model predicts that FtsZ accumulates faster when nutrient availability occurs more frequently. Because nutrient timescale and quantity were coupled in this study, whether different fluctuating timescales that deliver equal amounts of nutrient could enable different rates of FtsZ accumulation remains to be determined. Such empirical validation would support a mechanism of protein accumulation, or the levels of other macromolecules such as glycogen (Sekar *et al*. 2020), as a physiological mechanism by which cells sense and respond to different fluctuating timescales.

## OUTLOOK: EXPANDING OUR UNDERSTANDING OF MICROBIAL RESPONSES TO ENVIRONMENTAL FLUCTUATIONS

Whenever we try to understand a microbial process in nature, we ought to pay careful attention to the temporal component of environmental variability, as these fluctuations have major qualitative and quantitative effects on diverse processes. At every organizational level of microbial life, studies directly focused on how fluctuations affect microbial behavior have found diverse and often remarkable examples of microbial strategies that have evolved for life in dynamic environments. Similarly, studies in fluctuating environments have found timescale-dependent microbial responses. Environmental fluctuations can determine single-cell growth rates (Nguyen *et al*. 2020), the fixation of an allele in a population (Lin and Kussell 2016) and a community's metabolic output (DeAngelis *et al*. 2010) based on the relative timescale between environmental fluctuations and microbial response.

Studies that have successfully characterized an effect of fluctuations on a microbial process have an understanding for the timescales of that process. One important approach has been to shift microbes from one steady condition to another (Kjeldgaard, Maaløe and Schaechter 1958; Baumann *et al*. 1996; Schreiber *et al*. 2016; Erickson *et al*. 2017), mimicking one single shift within a sequence of fluctuations and measuring the timescale required for the microbial community, population or single cell to stabilize. While recent work has demonstrated that some responses to fluctuations fundamentally differ from responses to single shifts (Nguyen *et al*. 2020), still many responses have been elucidated from single-shift experiments. Similarly, a pulse in the environment can determine the timescale of processes such as species colonization or extinction: processes with effects that persist even after the environment has returned to the pre-pulse condition (Fig. 4C). For example, a 6-day period of increased gut osmolality induced the extinction of some abundant bacterial species, producing a new community composition that persist even after normal conditions returned (Tropini *et al*. 2018). Observing colonization is more difficult. A 5-day pulse of porphyran in mouse diets enabled an introduced porphyran-degrading *Bacteroides* strain to maintain a stable abundance in the gut for 10 days after the privileged nutrient was removed (Shepherd *et al*. 2018). From this, we learn that the stability in *Bacteroides* abundance outlasts the pulse by several days. However, we cannot determine if this colonization decays in weeks, months or ever.

Determining the timescale of a microbial response to fluctuations generates hypotheses for the biological mechanism underlying it. The remarkably consistent 1-cm spacing between all conical stromatolites formed in the last 2.8 billion years is now understood to be due to daily fluctuations in the metabolic activities of photosynthetic microorganisms (Petroff *et al*. 2010), a coupling between environmental and microbial fluctuations that have remained unchanged by time. Similarly, the type of response also provides mechanistic clues. *E. coli* has been observed to evolve generalist or specialist nutrient consumption strategies under nutrient fluctuations, depending on nutrients fluctuated (Sandberg *et al*. 2017). Generalists evolved under glucose/glycerol or glucose/xylose fluctuations, and subpopulations of specialist evolved under glucose/acetate fluctuations, suggesting that the pre-existing structure of metabolic networks may favor the emergence of certain strategies given specific sources.

Determining the timescale of a microbial response to fluctuations can also generate hypotheses for the types of environmental fluctuations for which it is adapted. Sporulation, a stochastic process by which growing *Bacillus subtilis* cells can differentiate into cells that are competent for DNA uptake (Süel *et al*. 2006) or into non-growing spores (Veening *et al*. 2008b), has been demonstrated to increase fitness in environments that fluctuate between nutrient availability and starvation (Siebring *et al*. 2014). Another form of bet-hedging strategy, the ability to sporulate is suggestive of unpredictable and challenging fluctuations, such as those in soils where up to 95% of microbial cells are estimated to be dormant at any given time (Jones and Lennon 2010; Couradeau *et al*. 2019). Similarly, it has been proposed that, in bacterial chemotaxis, the length of time over which the chemoattractant signal is time-averaged may have evolved in response to environmental timescales, allowing bacteria to sense changes in ligand concentration at a timescale relevant to affect swimming direction (Mora and Nemenam 2019). Finally, the diverse foraging strategies observed in different bacteria suggest that some may encounter higher fluctuation frequencies than others (Yawata *et al*. 2014; Smriga *et al*. 2016).

Overall, environmental fluctuations affect microbial physiology, ecology and evolution and thus have important implications to microbial ecosystems. To quantitatively bridge the microbial responses reviewed here with ecosystem dynamics, an important future direction will be to develop methods to characterize complex community dynamics under faster fluctuations (i.e. minute-scale). In the lab, combining the chemostat cultivation and monitoring of communities (Carrero-Colón, Nakatsu and Konopka 2006) with previously established techniques to dynamically measure gene expression and metabolite production (Baumann *et al*. 1996, 1997) would provide key insights into how communities respond to environmental fluctuations on these timescales. In the lab and in the field, studies should also note the differences in mRNA and protein timescales when planning measurements of gene expression (Moran *et al*. 2013) and consider selecting for triphosphate mRNA, to avoid also measuring degradation targeted monophosphate transcripts (Celesnik, Deana and Belasco 2007), which have slower half-lives. Finally, the majority of our knowledge on bacterial responses comes from fast-growing, easily cultured organisms. However, many ecosystems are dominated by slow-growing, less studied organisms, with fundamentally different responses to environmental change. Determining what fluctuations elicit which responses in these organisms will provide an important missing element in our understanding of microbial processes in their natural habitats.

## ACKNOWLEDGMENTS

The authors are grateful to Francesco Carrara, Johannes Keegstra, Russell Naisbit and Jeanette Wheeler for comments on our manuscript. We also thank our reviewers and editor for critical feedback that enhanced this work, and Cherry Gao and Ulrike Pfreundt for informative discussions.

## Supplementary Material

fuaa068_Supplemental_File

## References

[bib1] Acar M, Mettetal JT, Van Oudenaarden A. Stochastic switching as a survival strategy in fluctuating environments. Nat Genet. 2008;40:471. 18362885 10.1038/ng.110

[bib2] Ackermann M. A functional perspective on phenotypic heterogeneity in microorganisms. Nat Rev Microbiol. 2015;13:497–508. 26145732 10.1038/nrmicro3491

[bib3] Ahmerkamp S, Winter C, Janssen F et al. The impact of bedform migration on benthic oxygen fluxes. J Geophys Res-Biogeo. 2015;120:2229–42.

[bib7_856_1609813772530] Arevalo P, VanInsberghe D, Elsherbini J et al. A Reverse Ecology Approach Based on a Biological Definition of Microbial Populations. Cell. 2019;178:820–34. 31398339 10.1016/j.cell.2019.06.033

[bib4] Azam F, Malfatti F. Microbial structuring of marine ecosystems. Nat Rev Microbiol. 2007;5:782–91. 17853906 10.1038/nrmicro1747

[bib5] Balaban NQ, Merrin J, Chait R et al. Bacterial persistence as a phenotypic switch. Science. 2004;305:1622–5. 15308767 10.1126/science.1099390

[bib6] Baumann B, Snozzi M, Zehnder AJ et al. Dynamics of denitrification activity of *Paracoccus denitrificans* in continuous culture during aerobic-anaerobic changes. J Bacteriol. 1996;178:4367–74. 8755862 10.1128/jb.178.15.4367-4374.1996 PMC178201

[bib9_727_1609824335948] Baumann B, van der Meer JR, Snozzi M et al. Inhibition of denitrification activity but not of mRNA induction in Paracoccus denitrificans by nitrite at a suboptimal pH. Antonie van Leeuwenhoek. 1997;72:183–189. 9403103 10.1023/a:1000342125891

[bib7] Beaumont HJ, Gallie J, Kost C et al. Experimental evolution of bet hedging. Nature. 2009;462:90–3. 19890329 10.1038/nature08504

[bib8] Becker KW, Harke MJ, Mende DR et al. Combined pigment and metatranscriptomic analysis reveals synchronized diel patterns of phenotypic light response across domains in the open ocean. ISME J. 2020. 10.1038/s41396-020-00793-x PMC8027880 33033374

[bib10] Billerbeck M, Werner U, Polerecky L et al. Surficial and deep pore water circulation governs spatial and temporal scales of nutrient recycling in intertidal sand flat sediment. Mar Ecol Prog Ser. 2006;326:61–76.

[bib11] Blackburn N, Fenchel T, Mitchell J. Microscale nutrient patches in planktonic habitats shown by chemotactic bacteria. Science. 1998; 282:2254–6. 9856947 10.1126/science.282.5397.2254

[bib12] Blöschl G, Hall J, Parajka J et al. Changing climate shifts timing of European floods. Science. 2017;357:588–90. 28798129 10.1126/science.aan2506

[bib9] Boedicker JQ, Vincent ME, Ismagilov RF. Microfluidic confinement of single cells of bacteria in small volumes initiates high‐density behavior of quorum sensing and growth and reveals its variability. Angew Chem Int Ed. 2009;48:5908–11. 10.1002/anie.200901550 PMC2748941 19565587

[bib14] Carmody RN, Gerber GK, Luevano Jr JM et al. Diet dominates host genotype in shaping the murine gut microbiota. Cell Host Microbe. 2015;17:72–84. 25532804 10.1016/j.chom.2014.11.010 PMC4297240

[bib15] Carrero-Colón M, Nakatsu CH, Konopka A. Microbial community dynamics in nutrient-pulsed chemostats. FEMS Microbiol Ecol. 2006;57:1–8. 16819944 10.1111/j.1574-6941.2006.00095.x

[bib16] Celesnik H, Deana A, Belasco JG. Initiation of RNA decay in *Escherichia coli*by 5′ pyrophosphate removal. Mol Cell. 2007;27:79–90. 17612492 10.1016/j.molcel.2007.05.038 PMC2196405

[bib17] Chesson P. Mechanisms of maintenance of species diversity. Annu Rev Ecol Evol Syst. 2000;31:343–66.

[bib18] Chuang JS, Rivoire O, Leibler S. Simpson's paradox in a synthetic microbial system. Science. 2009;323:272–5. 19131632 10.1126/science.1166739

[bib19] Cohen SE, Golden SS. Circadian rhythms in cyanobacteria. Microbiol Mol Biol Rev. 2015;79:373–85. 26335718 10.1128/MMBR.00036-15 PMC4557074

[bib21] Cooper TF, Lenski RE. Experimental evolution with*E. coli*in diverse resource environments. I. Fluctuating environments promote divergence of replicate populations. BMC Evol Biol. 2010;10:11. 20070898 10.1186/1471-2148-10-11 PMC2827396

[bib20] Cooper VS, Lenski RE. The population genetics of ecological specialization in evolving *Escherichia coli* populations. Nature. 2000;407:736–9. 11048718 10.1038/35037572

[bib6_552_1609812857466] Coronado E, Valtat A, van der Meer JR. Sphingomonas wittichii RW 1 gene reporters interrogating the dibenzofuran metabolic network highlight conditions for early successful development in contaminated microcosms. Environmental Microbiology Reports. 2015;7:480–8. 25683238 10.1111/1758-2229.12276

[bib22] Couradeau E, Sasse J, Goudeau D et al. Probing the active fraction of soil microbiomes using BONCAT-FACS. Nat Commun. 2019;10:2710. 31235780 10.1038/s41467-019-10542-0 PMC6591230

[bib23] Cremer J, Segota I, Yang CY et al. Effect of flow and peristaltic mixing on bacterial growth in a gut-like channel. Proc Natl Acad Sci. 2016;113:11414–9. 27681630 10.1073/pnas.1601306113 PMC5068270

[bib24] Csonka LN. Physiological and genetic responses of bacteria to osmotic stress. Microbiol Mol Biol Rev. 1989;53:121–47. 10.1128/mr.53.1.121-147.1989 PMC372720 2651863

[bib13] Cárcamo-Oyarce G, Lumjiaktase P, Kümmerli R et al. Quorum sensing triggers the stochastic escape of individual cells from *Pseudomonas putida* biofilms. Nat Commun. 2015;6:1–9. 10.1038/ncomms6945 PMC4309448 25592773

[bib25] DeAngelis KM, Silver WL, Thompson AW et al. Microbial communities acclimate to recurring changes in soil redox potential status. Environ Microbiol. 2010;12:3137–49. 20629704 10.1111/j.1462-2920.2010.02286.x

[bib26] Dechesne A, Wang G, Gülez G et al. Hydration-controlled bacterial motility and dispersal on surfaces. Proc Natl Acad Sci. 2010;107:14369–72. 20660312 10.1073/pnas.1008392107 PMC2922541

[bib1_904_1609787645049] Desai MS, Seekatz AM, Koropatkin NM et al. A Dietary Fiber-Deprived Gut Microbiota Degrades the Colonic Mucus Barrier and Enhances Pathogen Susceptibility. Cell. 2016;167:1339–53. 27863247 10.1016/j.cell.2016.10.043 PMC5131798

[bib28] Donaldson GP, Ladinsky MS, Yu KB et al. Gut microbiota utilize immunoglobulin A for mucosal colonization. Science. 2018;360:795–800. 29724905 10.1126/science.aaq0926 PMC5973787

[bib27] Donaldson GP, Lee SM, Mazmanian SK. Gut biogeography of the bacterial microbiota. Nat Rev Microbiol. 2016;14:20–32. 26499895 10.1038/nrmicro3552 PMC4837114

[bib29] Elena SF, Lenski RE. Evolution experiments with microorganisms: the dynamics and genetic bases of adaptation. Nat Rev Genet. 2003;4:457–69. 12776215 10.1038/nrg1088

[bib30] Erickson DW, Schink SJ, Patsalo V et al. A global resource allocation strategy governs growth transition kinetics of *Escherichia coli*. Nature. 2017;551:119–23. 29072300 10.1038/nature24299 PMC5901684

[bib31] Fenchel T. Microbial behavior in a heterogeneous world. Science. 2002;296:1068–71. 12004118 10.1126/science.1070118

[bib33] Gayer CP, Basson MD. The effects of mechanical forces on intestinal physiology and pathology. Cell Signal. 2009;21:1237–44. 19249356 10.1016/j.cellsig.2009.02.011 PMC2715958

[bib34] Golden SS, Ishiura M, Johnson CH et al. Cyanobacterial circadian rhythms. Annu Rev Plant Biol. 1997;48:327–54. 10.1146/annurev.arplant.48.1.327 15012266

[bib36] Graham PJ, Nguyen B, Burdyny T et al. A penalty on photosynthetic growth in fluctuating light. Sci Rep. 2017;7:12513. 28970553 10.1038/s41598-017-12923-1 PMC5624943

[bib37] Guseva K, Feudel U. Numerical modelling of the effect of intermittent upwelling events on plankton blooms. J R Soc Interface. 2020;17:20190889. 32343934 10.1098/rsif.2019.0889 PMC7211471

[bib35] Görke B, Stülke J. Carbon catabolite repression in bacteria: many ways to make the most out of nutrients. Nat Rev Microbiol. 2008;6:613–24. 18628769 10.1038/nrmicro1932

[bib38] Hardcastle JD, Mann CV. Study of large bowel peristalsis. Gut. 1968;9:512. 5717099 10.1136/gut.9.5.512 PMC1552760

[bib39] Heisler J, Glibert PM, Burkholder JM et al. Eutrophication and harmful algal blooms: a scientific consensus. Harmful Algae. 2008;8:3–13. 28781587 10.1016/j.hal.2008.08.006 PMC5543702

[bib3_331_1609810027605] Hoces D, Arnoldini M, Diard M et al. Growing, evolving and sticking in a flowing environment: understanding IgA interactions with bacteria in the gut. Immunology. 2020;159:52–62. 31777063 10.1111/imm.13156 PMC6904610

[bib40] Hutchinson GE. The paradox of the plankton. Am Nat. 1961;95:137–45.

[bib41] Ishiura M, Kutsuna S, Aoki S et al. Expression of a gene cluster kaiABC as a circadian feedback process in cyanobacteria. Science. 1998;281:1519–23. 9727980 10.1126/science.281.5382.1519

[bib42] Jiménez‐Martínez J, Le Borgne T, Tabuteau H et al. Impact of saturation on dispersion and mixing in porous media: photobleaching pulse injection experiments and shear‐enhanced mixing model. Water Resour Res. 2017;53:1457–72.

[bib43] Jones SE, Lennon JT. Dormancy contributes to the maintenance of microbial diversity. Proc Natl Acad Sci. 2010;107:5881–6. 20231463 10.1073/pnas.0912765107 PMC2851880

[bib44] Kearney SM, Gibbons SM, Erdman SE et al. Orthogonal dietary niche enables reversible engraftment of a gut bacterial commensal. Cell Rep. 2018;24:1842–51. 30110640 10.1016/j.celrep.2018.07.032 PMC6724203

[bib45] Kim MK, Ingremeau F, Zhao A et al. Local and global consequences of flow on bacterial quorum sensing. Nat Microbiol. 2016;1:1–5. 10.1038/nmicrobiol.2015.5 PMC5010089 27571752

[bib46] Kjeldgaard NO, Maaløe O, Schaechter M. The transition between different physiological states during balanced growth of *Salmonella typhimurium*. Microbiology. 1958;19:607–16. 10.1099/00221287-19-3-607 13611203

[bib47] Kotte O, Volkmer B, Radzikowski JL et al. Phenotypic bistability in *Escherichia coli*'s central carbon metabolism. Mol Syst Biol. 2014;10. 10.15252/msb.20135022 PMC4299493 24987115

[bib48] Kussell E, Kishony R, Balaban NQ et al. Bacterial persistence: a model of survival in changing environments. Genetics. 2005;169:1807–14. 15687275 10.1534/genetics.104.035352 PMC1449587

[bib49] Kuzyakov Y, Blagodatskaya E. Microbial hotspots and hot moments in soil: concept & review. Soil Biol Biochem. 2015;83:184–99.

[bib50] Lambert G, Kussell E. Memory and fitness optimization of bacteria under fluctuating environments. PLos Genet. 2014;10:e1004556. 25255314 10.1371/journal.pgen.1004556 PMC4177670

[bib51] Leveau JH, König F, Füchslin H et al. Dynamics of multigene expression during catabolic adaptation of *Ralstonia eutropha* JMP134 (pJP4) to the herbicide 2, 4‐dichlorophenoxyacetate. Mol Microbiol. 1999;33:396–406. 10411755 10.1046/j.1365-2958.1999.01483.x

[bib52] Lin WH, Kussell E. Complex interplay of physiology and selection in the emergence of antibiotic resistance. Curr Biol. 2016;26:1486–93. 27212408 10.1016/j.cub.2016.04.015 PMC4899102

[bib53] Lodge DJ, McDowell WH, McSwiney CP. The importance of nutrient pulses in tropical forests. Trends Ecol Evol. 1994;9:384–7. 21236898 10.1016/0169-5347(94)90060-4

[bib55] Lozupone CA, Stombaugh JI, Gordon JI et al. Diversity, stability and resilience of the human gut microbiota. Nature. 2012;489:220–30. 22972295 10.1038/nature11550 PMC3577372

[bib54] López-Maury L, Marguerat S, Bähler J. Tuning gene expression to changing environments: from rapid responses to evolutionary adaptation. Nat Rev Genet. 2008;9:583–93. 18591982 10.1038/nrg2398

[bib56] Marchant HK, Ahmerkamp S, Lavik G et al. Denitrifying community in coastal sediments performs aerobic and anaerobic respiration simultaneously. ISME J. 2017;11:1799–812. 28463234 10.1038/ismej.2017.51 PMC5520038

[bib57] Maresca JA, Keffer JL, Hempel PP et al. Light modulates the physiology of nonphototrophic Actinobacteria. J Bacteriol. 2019;201:e00740–18. 30692175 10.1128/JB.00740-18 PMC6482932

[bib58] Melbinger A, Vergassola M. The impact of environmental fluctuations on evolutionary fitness functions. Sci Rep. 2015;5:15211. 26477392 10.1038/srep15211 PMC4609966

[bib59] Mitchell A, Romano GH, Groisman B et al. Adaptive prediction of environmental changes by microorganisms. Nature. 2009;460:220–4. 19536156 10.1038/nature08112

[bib5_396_1609810374961] Moor K, Diard M, Sellin ME et al. High-avidity IgA protects the intestine by enchaining growing bacteria. Nature. 2017;544:498–502. 28405025 10.1038/nature22058

[bib61] Moran MA, Satinsky B, Gifford SM et al. Sizing up metatranscriptomics. ISME J. 2013;7:237–43. 22931831 10.1038/ismej.2012.94 PMC3554401

[bib60] Mora T, Nemenman I. Physical limit to concentration sensing in a changing environment. Phys Rev Lett. 2019;123:198101. 31765216 10.1103/PhysRevLett.123.198101

[bib62] Morran LT, Schmidt OG, Gelarden IA et al. Running with the Red Queen: host-parasite coevolution selects for biparental sex. Science. 2011;333:216–8. 21737739 10.1126/science.1206360 PMC3402160

[bib63] Moxon ER, Rainey PB, Nowak MA et al. Adaptive evolution of highly mutable loci in pathogenic bacteria. Curr Biol. 1994;4:24–33. 7922307 10.1016/s0960-9822(00)00005-1

[bib64] Moxon R, Bayliss C, Hood D. Bacterial contingency loci: the role of simple sequence DNA repeats in bacterial adaptation. Annu Rev Genet. 2006;40:307–33. 17094739 10.1146/annurev.genet.40.110405.090442

[bib65] Muhkerjee S, Bassler BL. Bacterial quorum sensing in complex and dynamically changing environments. Nat Rev Microbiol. 2019;17:371–82. 30944413 10.1038/s41579-019-0186-5 PMC6615036

[bib66] Nguyen J, Fernandez V, Pontrelli S et al. A distinct growth physiology enhances bacterial growth under rapid nutrient fluctuations. BioRxiv. 2020; *under review at Nature Communications*. 10.1038/s41467-021-23439-8 PMC8209047 34135315

[bib2_490_1609789090389] Or D, Smets BF, Wraith JM et al. Physical constraints affecting bacterial habitats and activity in unsaturated porous media – a review. Advances in Water Resources. 2007;30:1505–27.

[bib67] Ottesen EA, Young CR, Eppley JM et al. Pattern and synchrony of gene expression among sympatric marine microbial populations. Proc Natl Acad Sci. 2013;110:E488–97. 23345438 10.1073/pnas.1222099110 PMC3568374

[bib68] Parulekar SJ, Semones GB, Rolf MJ et al. Induction and elimination of oscillations in continuous cultures of *Saccharomyces cerevisiae*. Biotechnol Bioeng. 1986;28:700–10. 18555381 10.1002/bit.260280509

[bib69] Paul BJ, Ross W, Gaal T et al. rRNA transcription in *Escherichia coli*. Annu Rev Genet. 2004;38:749–70. 15568992 10.1146/annurev.genet.38.072902.091347

[bib70] Pedrós-Alió C. Marine microbial diversity: can it be determined?. Trends Microbiol. 2006;14:257–63. 16679014 10.1016/j.tim.2006.04.007

[bib71] Pereira FC, Berry D. Microbial nutrient niches in the gut. Environ Microbiol. 2017;19:1366–78. 28035742 10.1111/1462-2920.13659 PMC5412925

[bib72] Petroff AP, Sim MS, Maslov A et al. Biophysical basis for the geometry of conical stromatolites. Proc Natl Acad Sci. 2010;107:9956–61. 20479268 10.1073/pnas.1001973107 PMC2890478

[bib73] Pett-Ridge J, Firestone MK. Redox fluctuation structures microbial communities in a wet tropical soil. Appl Environ Microbiol. 2005;71:6998–7007. 16269735 10.1128/AEM.71.11.6998-7007.2005 PMC1287741

[bib74] Placella SA, Brodie EL, Firestone MK. Rainfall-induced carbon dioxide pulses result from sequential resuscitation of phylogenetically clustered microbial groups. Proc Natl Acad Sci. 2012;109:10931–6. 22715291 10.1073/pnas.1204306109 PMC3390866

[bib75] Potts M. Desiccation tolerance of prokaryotes. Microbiol Mol Biol Rev. 1994;58:755–805. 10.1128/mr.58.4.755-805.1994 PMC372989 7854254

[bib76] Rainey PB, Beaumont HJ, Ferguson GC et al. The evolutionary emergence of stochastic phenotype switching in bacteria. Microb Cell Fact. 2011;10:S14. 21995592 10.1186/1475-2859-10-S1-S14 PMC3231921

[bib78] Rodríguez-Verdugo A, Ackermann M. Rapid evolution destabilizes species interactions in a fluctuating environment. ISME J. 2020:1–11. 10.1038/s41396-020-00787-9 PMC8027891 33024292

[bib77] Rodríguez‐Verdugo A, Vulin C, Ackermann M. The rate of environmental fluctuations shapes ecological dynamics in a two‐species microbial system. Ecol Lett. 2019;22:838–46. 30790416 10.1111/ele.13241

[bib79] Ross‐Gillespie A, Gardner A, Buckling A et al. Density dependence and cooperation: theory and a test with bacteria. Evolution. 2009;63:2315–25. 19453724 10.1111/j.1558-5646.2009.00723.x

[bib80] Rozen DE, Lenski RE. Long-term experimental evolution in *Escherichia coli*. VIII. Dynamics of a balanced polymorphism. Am Nat. 2000;155:24–35. 10657174 10.1086/303299

[bib81] Rozen DE, Philippe N, Arjan de Visser J et al. Death and cannibalism in a seasonal environment facilitate bacterial coexistence. Ecol Lett. 2009;12:34–44. 19019196 10.1111/j.1461-0248.2008.01257.x

[bib83] Sandberg TE, Lloyd CJ, Palsson BO et al. Laboratory evolution to alternating substrate environments yields distinct phenotypic and genetic adaptive strategies. Appl Environ Microbiol. 2017;83:e00410–17. 28455337 10.1128/AEM.00410-17 PMC5478979

[bib84] Schaechter M, Maaløe O, Kjeldgaard NO. Dependency on medium and temperature of cell size and chemical composition during balanced growth of *Salmonella typhimurium*. Microbiology. 1958;19:592–606. 10.1099/00221287-19-3-592 13611202

[bib85] Schreiber F, Littmann S, Lavik G et al. Phenotypic heterogeneity driven by nutrient limitation promotes growth in fluctuating environments. Nat Microbiol. 2016;1:16055. 27572840 10.1038/nmicrobiol.2016.55

[bib86] Scott M, Gunderson CW, Mateescu EM et al. Interdependence of cell growth and gene expression: origins and consequences. Science. 2010;330:1099–102. 21097934 10.1126/science.1192588

[bib88] Sekar K, Linker SM, Nguyen J et al. Bacterial glycogen provides short-term benefits in changing environments. Appl Environ Microbiol. 2020;86. 10.1128/AEM.00049-20 PMC7170487 32111592

[bib87] Sekar K, Rusconi R, Sauls JT et al. Synthesis and degradation of FtsZ quantitatively predict the first cell division in starved bacteria. Mol Syst Biol. 2018;14. 10.15252/msb.20188623 PMC6217170 30397005

[bib89] Seymour JR, Amin SA, Raina JB et al. Zooming in on the phycosphere: the ecological interface for phytoplankton–bacteria relationships. Nature Microbiology. 2017;2:17065. 10.1038/nmicrobiol.2017.65 28555622

[bib90] Sezonov G, Joseleau-Petit D, d'Ari R. *Escherichia coli* physiology in Luria–Bertani broth. J Bacteriol. 2007;189:8746–49. 17905994 10.1128/JB.01368-07 PMC2168924

[bib92] Shamir M, Bar-On Y, Phillips R et al. SnapShot: timescales in cell biology. Cell. 2016;164:1302. 26967295 10.1016/j.cell.2016.02.058

[bib93] Shamsuddin AM, Phelps PC, Trump BF. Human large intestinal epithelium: light microscopy, histochemistry, and ultrastructure. Hum Pathol. 1982;13:790–803. 7106744 10.1016/s0046-8177(82)80075-0

[bib91] Shepherd ES, DeLoache WC, Pruss KM et al. An exclusive metabolic niche enables strain engraftment in the gut microbiota. Nature. 2018;557:434–8. 29743671 10.1038/s41586-018-0092-4 PMC6126907

[bib94] Siebring J, Elema MJ, Vega FD et al. Repeated triggering of sporulation in *Bacillus subtilis* selects against a protein that affects the timing of cell division. ISME J. 2014;8:77–87. 23924781 10.1038/ismej.2013.128 PMC3869009

[bib8_904_1609818541702] Si F, Le Treut G, Sauls JT et al. Mechanistic origin of cell-size control and homeostasis in bacteria. Current Biology. 2019;29:1760–70. 31104932 10.1016/j.cub.2019.04.062 PMC6548602

[bib96] Smriga S, Fernandez VI, Mitchell JG et al. Chemotaxis toward phytoplankton drives organic matter partitioning among marine bacteria. Proc Natl Acad Sci. 2016;113:1576–81. 26802122 10.1073/pnas.1512307113 PMC4760798

[bib97] Stewart MK, Cummings LA, Johnson ML et al. Regulation of phenotypic heterogeneity permits Salmonella evasion of the host caspase-1 inflammatory response. Proc Natl Acad Sci. 2011;108:20742–7. 22143773 10.1073/pnas.1108963108 PMC3251048

[bib98] Stocker R. Marine microbes see a sea of gradients. Science. 2012;338:628–33. 23118182 10.1126/science.1208929

[bib99] Stocker R. The 100 µm length scale in the microbial ocean. Aquat Microb Ecol. 2015;76:189–94.

[bib82] Sæther BE, Engen S. The concept of fitness in fluctuating environments. Trends Ecol Evol. 2015;30:273–81. 25843273 10.1016/j.tree.2015.03.007

[bib100] Süel GM, Garcia-Ojalvo J, Liberman LM et al. An excitable gene regulatory circuit induces transient cellular differentiation. Nature. 2006;440:545–50. 16554821 10.1038/nature04588

[bib95] Słomka J, Alcolombri U, Secchi E et al. Encounter rates between bacteria and small sinking particles. New J Phys. 2020;22:043016.

[bib101] Tagkopoulus I, Liu YC, Tavazoie S. Predictive behavior within microbial genetic networks. Science. 2008;320:1313–7. 18467556 10.1126/science.1154456 PMC2931280

[bib102] Taylor JR, Stocker R. Trade-offs of chemotactic foraging in turbulent water. Science. 2012;338:675–9. 23118190 10.1126/science.1219417

[bib103] Tenaillon O, Barrick JE, Ribeck N et al. Tempo and mode of genome evolution in a 50,000-generation experiment. Nature. 2016;536:165–70. 27479321 10.1038/nature18959 PMC4988878

[bib107] Thaiss CA, Levy M, Korem T et al. Microbiota diurnal rhythmicity programs host transcriptome oscillations. Cell. 2016;167:1495–510. 27912059 10.1016/j.cell.2016.11.003

[bib106] Thaiss CA, Zeevi D, Levy M et al. Transkingdom control of microbiota diurnal oscillations promotes metabolic homeostasis. Cell. 2014;159:514–29. 25417104 10.1016/j.cell.2014.09.048

[bib104] Tropini C, Earle KA, Huang KC et al. The gut microbiome: connecting spatial organization to function. Cell Host & Microbe. 2017;21:433–42. 28407481 10.1016/j.chom.2017.03.010 PMC5576359

[bib105] Tropini C, Moss EL, Merrill BD et al. Transient osmotic perturbation causes long-term alteration to the gut microbiota. Cell. 2018;173:1742–54. 29906449 10.1016/j.cell.2018.05.008 PMC6061967

[bib109] Veening JW, Smits WK, Kuipers OP. Bistability, epigenetics, and bet-hedging in bacteria. Annu Rev Microbiol. 2008a;62:193–210. 18537474 10.1146/annurev.micro.62.081307.163002

[bib108] Veening JW, Stewart EJ, Berngruber TW et al. Bet-hedging and epigenetic inheritance in bacterial cell development. Proc Natl Acad Sci. 2008b;105:4393–8. 18326026 10.1073/pnas.0700463105 PMC2393751

[bib110] Vorholt JA. Microbial life in the phyllosphere. Nat Rev Microbiol. 2012;10:828–40. 23154261 10.1038/nrmicro2910

[bib111] Wolfson JS, Hooper DC, McHugh GL et al. Mutants of *Escherichia coli* K-12 exhibiting reduced killing by both quinolone and beta-lactam antimicrobial agents. Antimicrob Agents Chemother. 1990;34:1938–43. 1963289 10.1128/aac.34.10.1938 PMC171968

[bib112] Yawata Y, Cordero OX, Menolascina F et al. Competition–dispersal tradeoff ecologically differentiates recently speciated marine bacterioplankton populations. Proc Natl Acad Sci. 2014;111:5622–7. 24706766 10.1073/pnas.1318943111 PMC3992678

[bib113] Zarrinpar A, Chaix A, Yooseph S et al. Diet and feeding pattern affect the diurnal dynamics of the gut microbiome. Cell Metab. 2014;20:1006–17. 25470548 10.1016/j.cmet.2014.11.008 PMC4255146

