# Supplementary material for: Environmental fluctuations and their effects on microbial communities, populations and individuals
Source: FEMS Microbiol Rev. 2020 Dec 18;45(4):fuaa068. doi: 10.1093/femsre/fuaa068 (PMC8371271; doi:10.1093/femsre/fuaa068)
Supplement: fuaa068_Supplemental_File [file fuaa068_supplemental_file.docx]

Supplementary Table. An overview of the diversity of microbial responses to fluctuations discussed in our Review.

| **Fluctuation type** | **Organism, level** | **Timescale** | **Notes** | **Reference** |
| --- | --- | --- | --- | --- |
| Nutrient | Soil microbiota, all levels | Days - months | Evidence for microbial hotspots and hot moments in soil | Kuzyakov and Blagodatskaya (2015) |
| Nutrient | Soil microbiota, community and ecosystem | Days -months | The importance of nutrient pulses in tropical forests | Lodge et al. (1994) |
| Nutrient | Gut microbiome, all levels | Hours-days | Nutrient gradients in the gut | Pereira and Berry (2017) |
| Nutrient | Gut microbiome, community | 6 days | Fast returns to steady-state community composition after dietary change | Carmody et al. (2015) |
| Nutrient | Gut microbiome, community | 6 days | 8 hour shifts in meal times disrupts normal fluctuations in microbial community composition | Thaiss et al. (2014) |
| Nutrient | Gut microbiome, community | 4 days | Fluctuations enhance long-term engraftment of new species | Kearney et al. (2018) |
| Nutrient | Gut microbiome, community | Days | Timing of nutrient pulse can alter long-term community composition | Shepherd et al. (2018) |
| Nutrient (source) | *A. johnsonii & P. putida,* community | 2-10 days | Timescale of fluctuations determines whether both species are maintained in community | Rodríguez-Verdugo et al. (2019) |
| Nutrient (source) | *A. johnsonii & P. putida,* community | 2 days | Nutrient fluctuations resulted in the evolution of one species but not the other, disrupting species coexistence | Rodríguez-Verdugo and Ackermann (2020) |
| Nutrient | Freshwater and marine microbiota, community and populations | Days | Episodic or intermittent nutrient inputs simulate harmful algal blooms | Heisler et al. (2008); Guseva and Feudel (2020) |
| Nutrient | Gut microbiome, community | Hours | Fluctuations in host diet control fluctuations in gut community | Thaiss et al. (2014); Zarrinpar et al. (2014) |
| Nutrient | Gut microbiome, community | Hours | Fluctuations in microbial community control fluctuations in host gene expression | Thaiss et al. 2016 |
| Nutrient | *E. coli*, population | 2 days | Evolution of distinct ecotypes | Rozen et al. (2009) |
| Nutrient (source) | *E. coli*, population | 2 days | Greater genetic diversity evolves in fluctuating environments (vs. steady) | Cooper & Lenksi (2010) |
| Nutrient | Yeast, population | 1-3 days | Timescale of phenotypic switching can evolve based on timescale of environmental fluctuations | Acar et al. (2014) |
| Nutrient | *E. coli*, population | 6 days | Evolution of bet-hedging | Beaumont et al. (2009) |
| Nutrient | *E. coli*, population | Hours | Phenotypic split upon switch from glycolytic to gluconeogenic substrates | Kotte et al. (2014) |
| Nutrient (source) | *E. coli*, population | Hours-day | Experimental evolution of generalist or specialist strategies | Sandberg et al. (2017) |
| Nutrient | Yeast, population & single cell | Hours | Review on gene expression in changing environments | López-Maury et al. (2008) |
| Nutrient (source) | *S. typhimurium* or *E. coli*, single cell | Hours | Single shifts in nutrient source reveal steady-state model response | Kjeldgaard et al. (1958); Erickson et al. (2017) |
| Nutrient (source) | *E. coli* and *S. cerevisiae*, single cell | Hours | Anticipation of coupled nutrient shifts | Mitchell et al. (2009) |
| Nutrient (source) | *E. coli*, single cell | Minutes-hours | Cells develop shorter lag times after repeated fluctuations between glucose and lactose | Lambert and Kussell (2014) |
|  |  |  |  |  |
| Nutrient | *E. coli*, single cell | Minutes; hours | Potential mechanisms by which E. coli regulate cell division | Sekar et al. (2018); Si et al. (2019) |
| Nutrient | E. coli, single cell | Minutes | Potential mechanisms by which cells transition between starvation and growth in nutrient | Sekar et al. (2018); Sekar et al. (2020) |
| Nutrient | Marine microbiota, single cells | 10 minutes | Evidence for microscale hotspots | Blackburn et al. (1998); Fenchel (2002); Smriga and Fernandez et al. (2016) |
| Nutrient | *V. cyclitrophicus*, single cells | Minutes | Different wild isolates of *V. cyclitrophicus* use diverse foraging strategies | Yawata et al. (2014) |
| Nutrient | Marine microbiota, single cells | Seconds - minutes | Microbial length scales imply rapid fluctuating timescales | Stocker (2012); Stocker (2015) |
| Nutrient | *E. coli*, single cell | Seconds - minutes | Rapid fluctuations induce a fluctuation-adapted growth physiology | Nguyen et al., *under review* |
| Nutrient | Theory, single cell | Seconds | Theoretical limits on environmental sensing | Mora & Nemenam (2019) |
| Nutrient and light | Marine microbiota, community | Hours-day | Fluctuations in the gene expression of photosynthetic and heterotrophic bacteria | Ottesen et al. (2014); Becker et al. (2020) |
| Light | Actinobacteria, single cell | N.A. | Possible anticipation of higher nutrient concentrations by sensing light | Maresca et al. (2019) |
| Light | Cyanobacteria, single cell | 1 day | Anticipation of diurnal cycles via circadian rhythms | Golden et al. (1997); Ishiura et al. (1998); Cohen & Golden (2004) |
| Light | *Synechococcus elongatus* and alga *Chlamydomonas reinhardtii*, single cell | 10–100s | Light fluctuations reduce the growth rate of photosynthetic microbes in a timescale dependent manner | Graham et al. (2017) |
| Light | Stromatolite forming microbes in hot springs | 20 h | Spacing between stromatolites corresponds to a daily rhythm in photosynthetic metabolism | Petroff et al. (2010) |
| Oxygen | Soil microbial community | Days to seasons | Measurements of O_2_ fluctuations in soil | Silver et al. (1999) |
| Oxygen | Soil microbial community | 4 days | Fluctuation-adapted community is more active under fluctuating vs. static conditions | DeAngelis et al. (2010) |
| Oxygen | Soil microbial community | Daily-8 days | Incubated communities exposed to fluctuations on the timescale of days represent natural communities better than incubations in static or hour-scale fluctuating conditions | Pett-Ridge et al. (2005) |
| Oxygen | Marine sediment community | Minutes-hours | Metabolism deviates from steady-state model | Ahmerkamp et al. (2015); Billerbeck et al. (2006); Marchant et al. (2017) |
| Oxygen | Gut microbial community | Hours-days | Spatial gradients in the human gut | Tagkopoulus et al. (2008); Donaldson et al. 2016 |
| Oxygen | Marine bacteria, single cells | Minutes | Evidence for microscale hotspots | Fenchel (2002) |
|  |  |  |  |  |
| Hydration | *P. putida*, single cell | Minutes-hours | Motility is a stable trait amidst fluctuations in water availability | Dechesne et al. (2010) |
|  |  |  |  |  |
| Antibiotics | *E. coli*, populations and single cells | Minutes to hours | Strain that switches between susceptible and resistant mimics fixation of resistance in a population | Lin and Kussell (2016) |
| Antibiotics | *E. coli*, populations | Hours | About 1:10^5^ wild-type cells are persisters | Wolfson et al. (1990) |
| Antibiotics | *E. coli*, populations | Hours | Persistence as a phenotypic switch | Balaban et al. (2004) |
| Antibiotics | *E. coli*, populations | Hours | Theory on persistence | Kussell et al. (2005) |
|  |  |  |  |  |
| Host immune defenses | *H. influenza* and 8 additional human pathogens, populations | Days (every 100-1000 generations) | Genotype switches as a bet-hedging strategy | Moxon et al. (2006) |
| Host immune defenses | *S. typhimurium*, populations | Minutes-hours | Bistability of flagellar gene expression as a bet-hedging strategy | Stewart et al. (2011) |
|  |  |  |  |  |
| Temperature | *E. coli* and 8 other bacteria, populations | Hours | Populations evolved under fluctuations between 20 and 40°C do not grow significantly differently than those evolved at steady 30°C | Saarinen et al. (2018) |
| Temperature | *E. coli*, single-cell | 80 min | Anticipatory responses | Tagkopoulus et al. (2008) |
|  |  |  |  |  |
| Theoretical | Community | Variable | Intermediate disturbance hypothesis | Chesson (2000) |
| Theoretical | Population | Variable | Reduced sensitivity to environmental fluctuations can increase evolutionary fitness | Melbinger and Vergassola (2015) |
|  |  |  |  |  |
| Osmolality | Gut microbiome, community | Days | Temporary shift leads to long-term changes community composition | Tropini et al. (2018) |
| Osmolality | *E. coli*, single cell | Minutes | Fluctuations do not affect growth rate | Rojas et al. (2014) |
|  |  |  |  |  |
| Fluid flow rate | Gut microbiome, community | Minutes | Peristaltic motion induces fluid mixing | Cremer et al. (2016); Gayer & Basson (2009); Hardcastle and Mann (1968) |
| Fluid flow rate | *S. aureus* & *V. cholera*, populations | Hours | Variation in flow alters distribution of quorum sensing | Kim et al. (2016); Muhkerjee and Bassler (2019) |
